# Molecular signatures of cell diversity modulated by long noncoding RNAs in the human fetal spinal cord

**DOI:** 10.1016/j.isci.2026.115399

**Published:** 2026-03-17

**Authors:** Nan Miao, Trevor Lee, Liying Chen, Hegan Zhang, Jing Wang, Julianne Sun, Jason Sun, Yongqiang Sha, Shi-Ying Huang, Tao Sun

**Affiliations:** 1 Center for Precision Medicine, Huaqiao University, Xiamen, Fujian 361021, China; 2 Department of Cell and Developmental Biology, Cornell University Weill Medical College, New York, NY 10065, USA; 3 School of Medicine and School of Biomedical Sciences, Huaqiao University, Xiamen, Fujian 361021, China; 4 QuanZhou Women’s and Children’s Hospital, Quanzhou, Fujian 362000, China; 5 Xiamen Institute of Technology Attached School, Xiamen, Fujian 361021, China; 6 College of Ocean Food and Biological Engineering, Jimei University, Xiamen, Fujian 361021, China

**Keywords:** genomics, neuroscience, developmental biology

## Abstract

The human spinal cord controls sophisticated sensory perception and motor response across its extensive length. However, the spatial and temporal regulation of the generation of various motor neurons (MNs) and glial cells remains obscure. Using single-cell RNA sequencing (scRNA-seq) and single-cell Stereo RNA sequencing (scStereo RNA-seq), we here investigated and annotated molecular signatures of three neural stem cell (NSC) lineages, MNs, astrocytes, and oligodendrocytes, in the human fetal spinal cord from gestational week 8 (GW8) to GW12. We also found that some long noncoding RNAs (lncRNAs) have overlapping or reciprocal expression patterns with their adjacent coding genes, which are enriched with the promoter, enhancer, and CCCTC-binding factor (CTCF) in the same cell cluster. Our study generated a rich spatial expression library of molecular diversity of lncRNAs and their adjacent genes in neurons and glia in the human fetal spinal cord.

## Highlights

• Three NSC lineages were annotated in human fetal spinal cords
• Most motor neurons and glia were derived from the NSC2 lineage
• lncRNAs exhibited similar expressions with adjacent coding genes
• The *cis* lncRNA-gene pairs were co-expressed in spinal neurons and glia

## Graphical abstract

**Figure:**
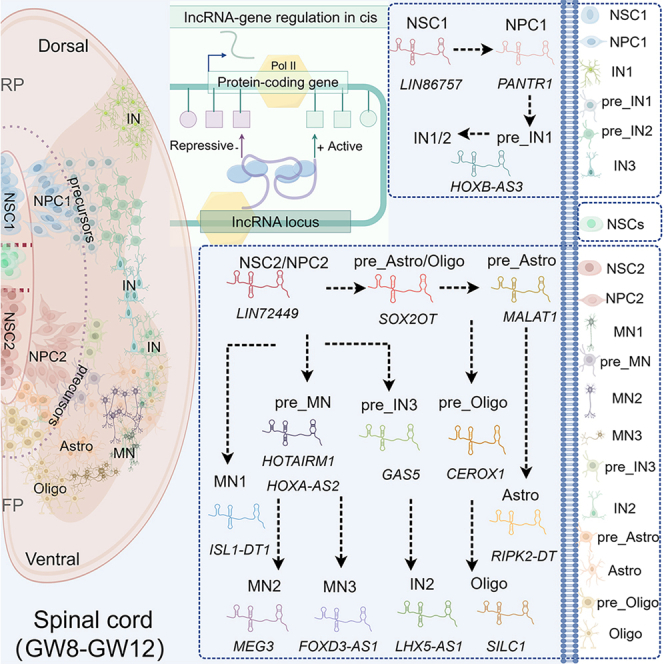

## Introduction

The spinal cord is positioned in the caudal segment of the mammalian central nervous system (CNS) and plays a crucial role in transmitting and processing neural circuit information between the brain and peripheral regions.^1^^,^^2^^,^^3^^,^^4^ During the spinal cord development, neural stem cells (NSCs) in the ventricular zone (VZ) lining to the central canal give rise to neural progenitor cells (NPCs) and glial progenitors in the subventricular zone (SVZ), and only a small population of NSCs maintains limited self-renewal capacity.^5^^,^^6^^,^^7^^,^^8^^,^^9^ NPCs in the dorsal and ventral spinal cord are further differentiated into various motor neurons (MNs) and interneurons (INs) to facilitate motor and sensory functions.^10^^,^^11^ Glial progenitors are differentiated into astrocytes and oligodendrocytes to modulate a dynamic spectrum of CNS development, maturation, and maintenance.^12^^,^^13^^,^^14^

In the vertebrate spinal cord, MNs are organized into distinct columns, each responsible for innervating specific peripheral domains. The median motor column (MMC) innervates axial muscles, the hypaxial motor column (HMC) innervates body wall muscles, and the lateral motor column (LMC) innervates limb musculature.^15^^,^^16^^,^^17^^,^^18^ The spinal sensory information, such as skin sensory input and rhythmic and flexible motor control, is facilitated by INs with 11 subtypes, including six dorsal INs (dIs and dI1-dI6), two late-born dorsal INs (dILA-B), and four ventral INs (V0-V3).^19^^,^^20^^,^^21^ Locomotion in the vertebrate is activated by a complex intraspinal neuron network that is decoded by descending MNs and afferent INs.^22^^,^^23^^,^^24^

Moreover, NSCs also are specified to switch cell fate and produce progenitors for astrocytes and oligodendrocytes.^25^ Spinal astrocyte precursors are sequentially generated from progenitors in an order from the ventral to dorsal spinal cord, while oligodendrocyte precursors are produced from progenitors in the ventral spinal cord.^26^^,^^27^ It appears that the spinal glial cells, once generated in the specific ventral or dorsal positions, seldom exhibit secondary tangential migration, even after acute CNS injury.^28^^,^^29^^,^^30^ Therefore, understanding the mechanisms for neuro-glia lineage switch is fundamental for unraveling how proper numbers of diverse neuronal and glial cell types are controlled.^13^^,^^31^^,^^32^^,^^33^ However, due to the long segmental structure of the human spinal cord, how various cell types are temporally and spatially organized and how distinct genes and long noncoding RNAs (lncRNAs) are orchestrated during fetal stages remain unclear.^34^^,^^35^

Because single-cell RNA sequencing (scRNA-seq) and single-cell Stereo RNA sequencing (scStereo RNA-seq) enable a comparative analysis of transcriptomes across thousands of individual cells, they have generated valuable molecular cell origin and lineage landscapes in the human fetal and adult CNS.^36^^,^^37^ Human spinal cord cell atlases in a spatial resolution are beginning to be revealed at fetal stages and adulthood.^3^^,^^6^^,^^38^^,^^39^^,^^40^^,^^41^^,^^42^^,^^43^ Interestingly, because scRNA-seq and scStereo RNA-seq can effectively produce RNA transcript information in individual cells, in particular lncRNAs, which are often underrepresented in bulk RNA sequencing due to their low abundance, they have been demonstrated to show specific spatiotemporal precision in distinct cell types in the human brain.^38^^,^^43^^,^^44^^,^^45^^,^^46^^,^^47^ Integrating temporal and cell type-specific expressions of both gene and lncRNA profiles will help build comprehensive molecular architectures of the human CNS.

To unravel molecular diversity and developmental trajectory of spinal neurons and glial cells in the human fetal spinal cord, we here conducted the scRNA-seq and scStereo RNA-seq, using spinal tissues from gestational week 8 (GW8), GW10, and GW12. Integration of these datasets revealed enriched expression of coding genes and lncRNAs in three clusters of NSCs and diverse MN and glial cell lineages. Some lncRNAs were located with adjacent coding genes in the human genome. Our findings provide a rich reference to further explore temporal and spatial regulations of the generation of diverse neuron and glial lineages in the human fetal spinal cord.

## Results

### Cell diversity and spatial domains in the human fetal spinal cord

**Figure 1:**
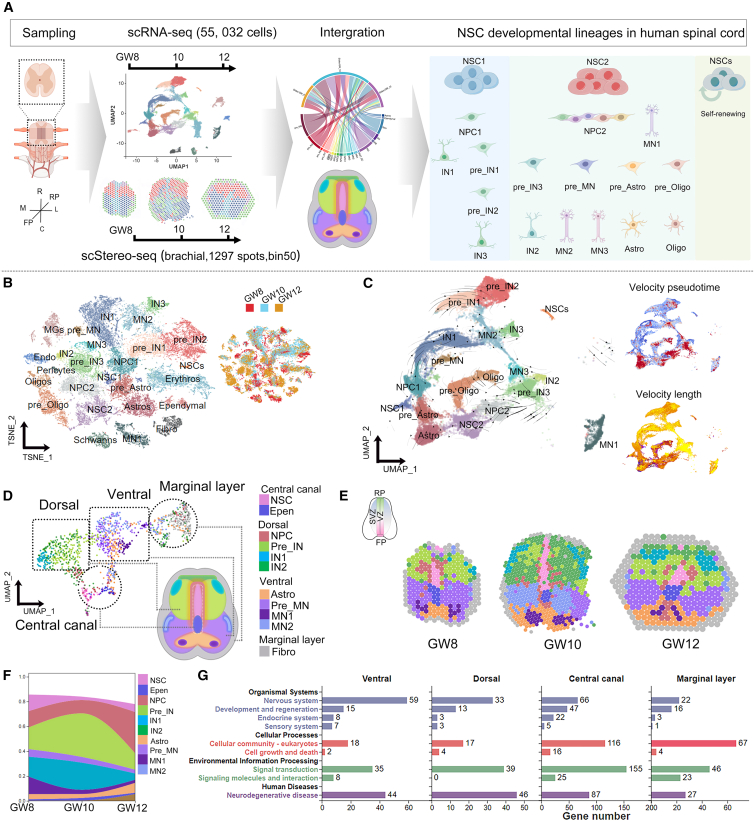
Spatially resolved cell cluster profiles in the human fetal spinal cord (A) The spatiotemporally resolved transcriptomic profiling of the developing human spinal cord across GW8-12 analysis was obtained using 10× Genomics scRNA-seq and scStereo RNA-seq technologies. This schematic provides an overview of the experimental design and analysis and three developmental trajectories in the spinal cord. (B) 27 distinct cell types were identified and annotated in the human fetal spinal cords from GW8 to GW12. (C) After excluding non-neural cells, 20 clusters with velocity were visualized using UMAP. (D and E) These scRNA-seq clusters were integrated into 11 scStereo RNA-seq clusters (GW8, GW10, and GW12), revealing four domains: central canal, ventral, dorsal, and marginal layers. (F) Calculation of the proportions of cell clusters. (G) KEGG analysis was conducted for each domain. The cell clusters are displayed in UMAP and slides with distinct colors: pink series for NSC and NPC clusters, green series for IN clusters, purple series for MN clusters, and orange series for Astro clusters. Spot: 100 μm diameter. UMAP, uniform manifold approximation and projection; NSC, neural stem cell; NPC, neural progenitor cell; IN, interneuron; MN, motor neuron.

To investigate the trajectory development and spatial organizations of NSCs to neurons and glial cells in the human fetal spinal cord, we conducted spatial transcriptomic and scRNA-seq analyses using human fetal tissues at GW8, GW10, and GW12. Transcriptional profiles of 55,032 single cells were captured from fetal spinal cords, using 10× Genomics scRNA-seq (Table S1), and spatially resolved transcriptomic data were obtained from slides of fetal spinal cords at the brachial level (1,297 spots, bin50), using 10× Visium atlas (Figure 1A). We employed single-cell transcriptomic data to visualize the cellular composition of each spatial location, utilizing the residual convolutional neural network with Transformer Decoder (RCTD), Stereoscope, and Cell2location tools.^48^^,^^49^^,^^50^

Based on the scRNA-seq data, we annotated coding and noncoding RNA transcripts in 55,032 cells and merged them with the reference genome *Homo sapiens* GRCh38.p14. We detected the accumulation of 37,899 reads and 3,993 UMIs per cell in average, and captured 20,484 coding genes and 19,606 lncRNAs across all sequenced cells (Tables S2 and S3). After excluding the cell cycle genes (Figure S1A), these cells were further categorized into 27 major clusters, including NSCs, NPCs, neural precursors, glial precursors, neurons, glial cells, and other cell types, as visualized using t-distributed stochastic neighbor embedding (t-SNE) and uniform manifold approximation and projection (UMAP) (Figures 1B, 1C, and S1B–S1D). Next, we analyzed neuronal and glial differentiation trajectories by using RNA velocity analysis, which revealed three NSC subtypes, including NSC1, NSC2, and NSCs in GW8, GW10, and GW12 (Figures 1C, S2A, and S2B; Tables S4, S5, S6, S7, and S8).^51^^,^^52^

We further identified four spatial domains in the human fetal spinal cord at GW8, GW10, and GW12, including the central canal, ventral, dorsal, and marginal layers, using scStereo RNA-seq. To explore the molecular traits and spatial arrangements of spinal neurons and glia, we analyzed the cell composition at each spot, using single-cell transcriptome data and detected the dominant cell clusters in each domain (Figures 1D, S2C, and S2D; Tables S9, S10, S11, S12, and S13). For example, the central canal contained 3 NSC and 1 ependymal (Epen) clusters, the dorsal region contained 1 NPC and 3 IN clusters, and the ventral region contained 3 MN clusters (Figure 1E and Tables S14, S15, S16, S17, and S18). To elucidate the timing of neurogenesis and gliogenesis in the human fetal spinal cord, we analyzed the distribution of various cell clusters from GW8 to GW12 and detected a reduction in the proportions of NSC and MN1 cells, accompanied by an increase in the proportions of NPC1, MN, and ependymal cells (Figure 1F). Notably, astrocytes were dominant at GW12, while precursors of interneuron (pre_IN) and IN2 cells were dominant at GW10. KEGG analyses further revealed that genes in the central canal were involved in the nervous systems, signal transduction and neurodegenerative diseases, genes in the ventral were more prevalent in the nervous system, genes in the dorsal were more active in signal transduction and neurodegenerative diseases, indicating the distinct functions for cell clusters in each region of the human fetal spinal cord (Figure 1G and Tables S19, S20, and S21).

In summary, our results of scRNA-seq and scStereo RNA-seq detected high expression of coding genes and noncoding RNA transcripts and demonstrated highly diverse cell types with their distinct spatial domains, which consist of genes of specific functional output, in the human fetal spinal cord.

### Trajectory development of NSCs in the human fetal spinal cord

**Figure 2:**
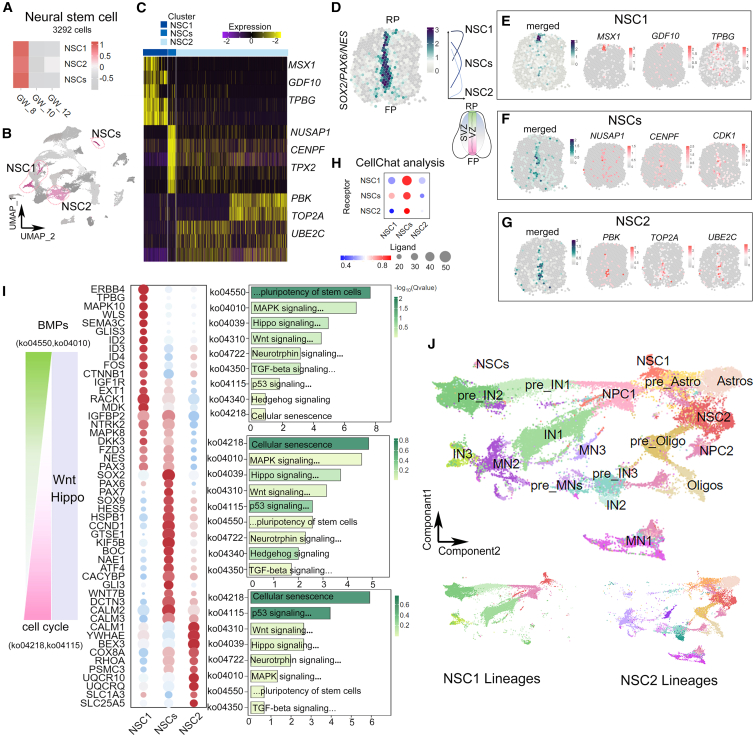
Cell clusters corresponding to the anatomical regions in 3 NSC lineages in the human fetal spinal cord (A and B) The development of three NSC clusters-NSC1, NSC2, and NSCs in the spinal cord peaks at GW8 via heatmap and visualized using UMAP. (C) Heatmaps showing *MSX1*/*GDF10*/*TPBG* in NSC1, *NUSAP1*/*CENPF*/*TPX2* in NSCs, and *PBK*/*TOP2A*/*UBE2* in NSC2 (yellow, high; black, median; yellow: low). (D) Common stem cell markers (*SOX2*, *PAX6*, and *NES*) were tested in these clusters within the central canal. (E–G) From the roof plate (RP) to the floot plate (FP), NSC1 (E), NSCs (F), and NSC2 (G) with specific markers were mapped using scStereo RNA-seq, with each 50 μm bin color coded for expression levels (co-expressed: green, high; white, median; gray, low; separated gene expression: red, high; white, median; gray, low). (H) Cellchat was used to identify ligand-receptor correlations within three NSC clusters. (I) Comparative KEGG analysis of BMPs, SHH, and cell cycles was conducted on differentially expressed genes in three NSC clusters. (J) Slingshot was used to map the developmental lineage switch of NSC2, which contributes to motor neuron, IN2, astrocyte, and oligodendrocyte differentiation. Spot: 100 μm diameter. NSC, neural stem cell; RP, roof plate; FP, floor plate; BMPs, bone morphogenetic proteins; SHH, sonic hedgehog.

To reveal trajectory development of 3 distinct NSC clusters (3,292 cells), we first analyzed gene expression profiles in them from GW8 to GW12 (Figure 2). Both common stem cell markers and unique gene expression combinations were detected in 3 NSC clusters (Figures 2A–2C, S3, S4A, and S4B; Table S22). Specifically, while *SOX2*, *PAX6*, and *NES* were identified in all 3 NSC clusters (Figure S4C), particularly in the central canal, *MSX1*, *GDF10*, and *TPBG* were detected in the NSC1, *NUSAP*/*CEPF*/*CDK1* in the NSC2, and *PBK*/*TOP2A*/*UBE2C* in the NSCs (Figure 2C). Moreover, the scStereo RNA-seq data showed that *MSX1*, *GDF10*, and *TPBG* in the NSC1 are mostly co-expressed in the dorsal region, *NUSAP1*, *CENPF*, and *CDK1* in the NSCs in the median dorsal-ventral axis, and *PBK, TOP2A*, and *UBE2C* in the NSC2 in the ventral region in the human fetal spinal cord at GW10 (Figures 2D–2G and S4C).

In addition, CellChat was used to identify cell communications and ligand-receptor (LR) interactions among 3 NSC clusters (Figures 2H and S5A). The results showed that NSCs communicate with NSC1 and NSC2 through MDK-PTPRZ1/NCL and PTN-NCL pairs, which functioned in the mitogen-activated protein kinase (MAPK) pathway (Figures 2H and S5B).^53^^,^^54^ Wingless-type family member 7B (WNT7B)-frizzled class receptor (FZD)-related pairs were detected as the most active LR interactions within the NSC cluster and functioned in bone morphogenetic proteins (BMPs) and WNT signal pathways, suggesting the potential interactions of three distinct NSC clusters (Figure S5C).^55^^,^^56^ Moreover, previous studies have shown that transcription factors (TFs) expressed along the spinal dorsal-ventral axis respond to signals such as BMPs from the roof plate (RP) and molecules such as sonic hedgehog (Shh) in the floor plate (FP).^57^^,^^58^^,^^59^^,^^60^ We next analyzed TFs expressed in the 3 NSC clusters and found that TFs associated with the MAPK and transforming growth factor β (TGF-β) pathways are dominant in the NSC1 in the dorsal spinal region, and those related to the p53 and cellular senescence pathways are prevalent in the NSC2 in the ventral spinal region (Figure 2I and Table S23). TFs related to the WNT and Hippo pathways maintained a stable expression level in 3 NSC clusters. These results suggest that sonic hedgehog (SHH), MAPK, and WNT signaling are essential to maintain the spinal NSCs.

Furthermore, we analyzed developmental trajectories of the 3 NSC clusters. We first annotated genes in the NSC clusters and detected *ERBB4*/*MAPK10*/*WLS* in the NSC1, *PAX7*/*CCND1*/*GTSE1* in the NSCs, and *CALM1*/*YWHAE*/*BEX3* in the NSC2 (Figure 2I). We then analyzed the NSC1 cluster and detected specific expression combinations of *GAD2*/*LHX2*/*TFAP2A* in the pre_IN1, *GRIA1*/*SYT1*/*PAX2* in the pre_IN2, and *SST*/*NELL1*/*PAX8* in the IN3, suggesting that the NSC1 cluster is strongly associated with the differentiation of the IN lineage (Figures 2J, S6, S7A, and S8; Tables S24 and S25) and the NSC2 with lineages of the MNs (MN1-MN3), IN2, and glia (astrocytes and oligodendrocytes) (Figures S6 and S7B), while the NSCs maintained the stem cell population (Figure 2J).

We next analyzed MNs and glial cells in the NSC2 trajectory (Figures S7B and S9). We annotated three MN columns by using known markers and identified the MMC enriched with the MN1 expressing *ISL1*/*PPP1R17*/*NEUROD1*, the HMC with the MN2 expressing *GAP43*/*SNCG*/*LHX3*, and the LMC with the MN3 expressing *LHX2*/*CBLN1*/*EVX1* (Figures S9A and S9B).^15^^,^^16^^,^^17^^,^^18^ Moreover, the pre_MN initiated differentiation to the MN2 and MN3 at GW8 and was detected at the same spatial location as the pre_IN3, which underwent differentiation into IN2 from the ventral to dorsal spinal cord at GW10 (Figure S9C). These results suggest that MNs and some populations of INs are derived from NSCs in the ventral VZ of the spinal cord.

We also detected combined expressions of *GLI3*, *TNC*, and *SOX9* in the astrocyte precursors (pre_Astro) and of *SLC6A9*, *SLC6A11*, and *FGFBP3* in the differentiated astrocytes (Astro), suggesting that the NSC2 as well gives rise to the Astro trajectory (Figure S9D). Moreover, expressions of *SOX5*, *OLIG1*, and *OLIG2* were detected in the oligodendrocyte precursors (pre_Oligo), and *PLP1*, *MBP*, and *SOX10* were annotated in the oligodendrocytes (Oligo), indicating an early commence of astrocytes and oligodendrocytes from the NSC2 cluster (Figure S9E). Moreover, spatial dots of the scStereo RNA-seq data showed that the Astro population is positioned from the ventral to dorsal domains in the human fetal spinal cord, while the Oligo lineage is restricted to the ventral domain (Figure S9F).

In summary, our scRNA-seq and scStereo RNA-seq datasets have identified specific molecular signatures of three spinal NSC populations in the distinct region of the central canal. While the NSC1 gives rise to the IN trajectory, the NSC2 mainly undergoes differentiations into MNs, Astro, and Oligo.

### Cell type-specific expression of lncRNAs in the human fetal spinal cord

One interesting observation from our scRNA-seq and scStereo RNA-seq data is that a significant number of lncRNAs was detected in various cell clusters. Because lncRNAs display specific expressions in distinct cells in the nervous system,^46^^,^^47^ we decided to investigate lncRNA regulations in each cell cluster.

**Figure 3:**
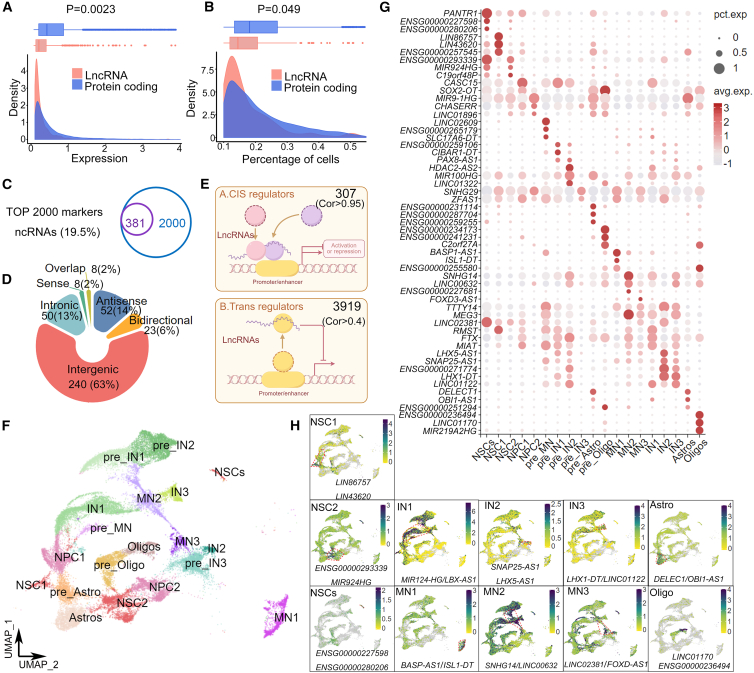
Cell clusters corresponding to anatomical regions in 3 NSC lineages in the human fetal spinal cord (A and B) The distribution of median gene expression levels and the percentage of cells expressing lncRNA (red) and protein-coding (blue) genes were analyzed using the Mann-Whitney U test. (C) Venn diagram shows that 19.5% (381) of the lncRNAs were tested among the top 2,000 markers. (D) In a donut plot, these lncRNAs are categorized into seven groups based on specific chromosome loci. (E) Correlation analysis within the same cluster identified 279 *cis* pairs (Cor > 0.95) and 3,919 *trans* pairs (Cor > 0.4). (F) UMAP visualization shows lncRNA markers in neuron and glial clusters in spinal cord scRNA-seq. (G) A bubble chart illustrates lncRNA contributions, with dot color and size indicating expression level and cell fraction. (H) lncRNAs served as cell markers: *LIN86757*/*LIN43620* in NSC1, *ENSG00000293339*/*MIR924HG* in NSC2, and *ENSG00000227589*/*ENSG00000280206* in NSCs; *BASP-AS1*/*ISL1-DT* in MN1, *SNHG14*/*LINC00632* in MN2, and *LINC02381*/*FOXD-AS1* in MN3; *RMST*/*FTX* in IN1, *SNAP25-AS1*/*LHX5-AS1* in IN2, and *LHX1-DT*/*LINC01122* in IN3; *DELEC1*/*OBI1-AS1* in Astro; and *LINC01170*/*ENSG00000236494* in Oligo (green, high; yellow, low).

We first compared the expression levels of protein-coding genes with lncRNAs and detected a lower expression of lncRNAs (*p* value = 0.0023) in human fetal spinal cells (Figure 3A). lncRNAs expressed in fewer spinal cell types with a narrow expression range (*p* value = 0.049) than protein-coding genes (Figure 3B). Moreover, we identified and annotated 381 lncRNAs, representing 19.5% of the top 2,000 highly expressed transcripts in human fetal spinal cells (Figure 3C). These lncRNAs were further classified into 6 types according to their genome locations, including 240 intergenic, 50 intronic, 52 antisense, 23 bidirectional, 8 sense, and 8 overlapped lncRNAs (Figure 3D). We next categorized these lncRNAs into *cis* (307, Cor > 0.95) and *trans* (3,919, Cor > 0.4) according to their potential regulatory roles to their adjacent genes (Figures 3E and 3F; Tables S26, S27, and S28).

We further annotated specific lncRNAs in distinct spinal cell clusters at GW8-GW12, for instance, *ENSG00000286757* (termed as *LIN86757*)/*ENSG00000243620* (termed as *LIN43620*) in the NSC1, *ENSG0000029339*/*MIR924HG* in the NSC2, *ENSG00000227598*/*ENSG00000280206* in the NSCs, *CASC15*/*LINC03000* in the NPC1, and *MIR9-1HG*/*CHASERR* in the NPC2, using both UMAP and spatial resolution (Figures 3G, 3H, S10, and S11). lncRNAs such as *RMST* and *FTX* were highly abundant in the IN1, *SNAP25-AS1* and *LHX5-AS1* in the IN2, and *LHX-DT* and *LINC01122* in the IN3 (Figures 3G, 3H and S10–S12). Moreover, lncRNAs such as *BASP-AS1* and *ISL1-DT* were highly expressed in the MN1, *SNHG14*/*LINC00632* in the MN2, and *LINC02381*/*FOXD3-AS1* in the MN3 (Figures 3G, 3H, S11B, and S13). lncRNAs *DELEC1* and *OBl1-AS1* were detected in the Astro, and *LINC01170* and *ENSG00000236494* were detected in the Oligo (Figures 3G, 3H, S11B, and S14). These results suggest that like protein-coding genes, lncRNAs display cell type-specific expression patterns in regulating NSC developmental trajectories in the human fetal spinal cord.

### Co-expression of lncRNAs and their adjacent coding genes in the human fetal spinal cord

**Figure 4:**
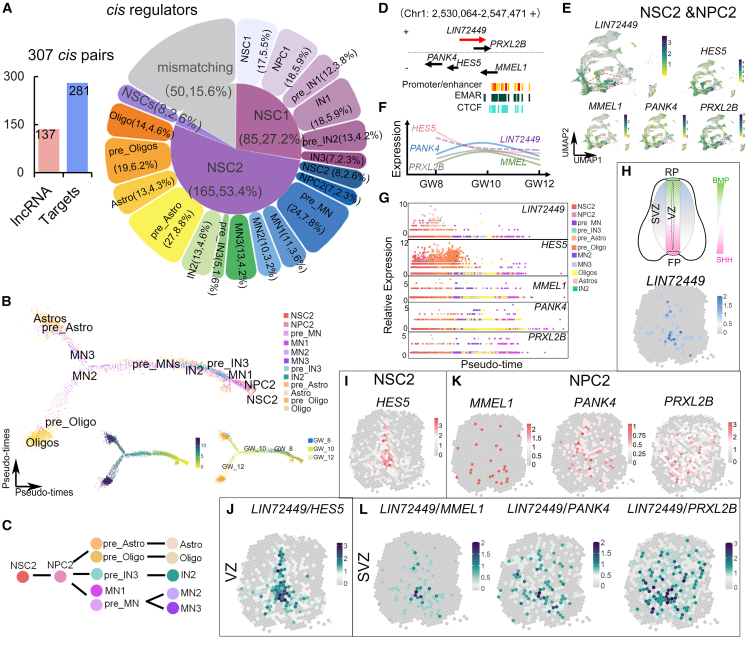
lncRNAs and their adjacent coding genes in the NSC2-NPC2 lineage in the human fetal spinal cord (A) Bar chart displaying 279 *cis* pairs among 137 lncRNAs and 274 targets, and a double pie chart illustrating *cis* regulators in NSC1 (83, 27.2%), NSC2 (165, 53.4%), and NSCs (8, 2.6%). (B) The NSC2 developmental trajectory analysis by Monolcle2. (C) NSC2 contributes to motor neuron, IN2, astrocyte, and oligodendrocyte differentiation. (D) The *cis* roles of *LIN72449* in NSC2 and NPC2 were examined, with 4 genes on chr1. (E) *LIN72449*, *HES5*, *PANK4*, *MMEL1*, and *PRXL2B* were visualized by UMAP (green, high; yellow, low). (F) The expression trends of *LIN72449* and 4 genes from GW8 to GW12. (G) *LIN72449*, *HES5*, *PANK4*, *MMEL1*, and *PRXL2B* with their pseudo-time trends, shown in a dot plot, separately. (H, I, and J) At GW10 spatial pattern, *LIN72449* (H), *HES5* (I) and co-expressed *LIN72449*/*HES5* (J) were exhibited in NSC2. (K and L) *PANK4, MMEL1, and PRXL2B* (K) and their co-expression with *LIN72449* (L) were exhibited in NPC2. Spot: 100 μm diameter.

One interesting phenomenon of lncRNA regulation is to form *cis*-regulatory pairs with adjacent coding genes in the genome and modulate neural development.^61^ We, thus, predicted that spinal lncRNAs might participate in lineage switch of NSCs to neurons and glia by forming pairs with their adjacent coding genes. We annotated scRNA-seq and scStereo RNA-seq data and identified 307 potential *cis*-regulatory lncRNA-coding gene pairs, comprising 137 lncRNAs and their 281 adjacent RNA transcripts in different clusters (Figure 4A). Among these pairs, 85 (27.2%) were identified in the NSC1 trajectory, 165 (53.4%) in the NSC2, and 8 (2.8%) in the NSCs. In the NSC1 developmental lineage, 17 (5.5%) lncRNA-coding gene pairs were detected in the NSC1 cluster itself, 18 (5.9%) in the NPC1, and 18 (5.9%) in the IN1. In the NSC2 developmental lineage, 24 (7.8%) pairs were identified in the pre_MN cluster, 27 (8.8%) in the pre_Astro, and 19 (6.2%) in the pre_Oligo (Figure 4A).

Moreover, several *cis*-regulatory pairs of lncRNA-coding genes were displayed in the NSC1 (Figures S15 and S16A). For instance, *LIN86757* displayed similar sequential expression levels to *ZIC2, ZIC5*, and *CLYBL* along the pseudo time from GW10 to GW12, with a higher expression in the NSC1 and NPC1 clusters and a lower expression in the IN clusters (Figure S16B). In addition, *LIN43620* and *ZIC1* displayed similar sequential expression levels, with a higher expression in the NSC1, NPC1, and pre_IN clusters and a lower expression in the IN clusters along the pseudo time from GW10 to GW12 (Figure S16). Furthermore, previously reported lncRNAs such as *PANTR1* and *HOXB-AS3*^38^^,^^62^^,^^63^ were also detected in IN sub-lineages (Figure S17). *HOXB-AS3* was broadly expressed in the dorsal SVZ in the human fetal spinal cord at GW10, which was like *HOXB7*, *HOXB2*, and *HOXB3* expression in the pre_IN1 cluster, *HOXB9* and *HOXB5* in the pre_IN2, and *HOXB6* and *HOXB8* in the IN1 (Figure S17). These results suggest that the enrichment of lncRNAs and their adjacent coding genes in each specific cell type might serve as a *cis*-regulatory manner to produce neurons and glia cells from NSCs in the human fetal spinal cord.

We observed that the developmental trajectory of the NSC2 lineage is to give rise to MNs and IN2 (Figures 4B and 4C), and one lineage is to generate astrocytes and oligodendrocytes (Figures 4B and 4C). We found a high expression of the lncRNA *ENSG00000272449*, termed as *LIN72449* in this study, in both NSC2 and NPC2 clusters (Figures 4D and 4E). *LIN72449* was located on the forward strand in the human chr1 p36.32, where a CCCTC-binding factor (CTCF) was enriched with promoter and enhancer elements (Figure 4D). Hes family bHLH transcription factor 5 (*HES5*) was positioned in a tail-to-tail orientation relative to *LIN72449,* with pantothenate kinase 4 (*PANK4,* also known as *CTRCT49*) located upstream, membrane metalloendopeptidase-like 1 (*MMEL1*, also known as *NEP2*) situated downstream, and peroxiredoxin-like 2B (*PRXL2B*, also known as *FAM213B*) overlapping with *LIN72449* on the reverse strand (Figure 4D). Moreover, we found that all these four genes display a similar abundance in the NSC2 cluster (Figure 4E), and *LIN72449* and *HES5* show declined expression levels during fetal development, with the highest expression at GW8 (Figures 4F, 4G, and S18A).

In the NSC2, *LIN72449* and *HES5* displayed similar expression patterns in the dorsal and ventral VZs in the human fetal spinal cord at GW10 based on the scStereo RNA-seq data (Figures 4H and 4I). We merged spatial expression dots of *LIN72449* and *HES5* and found that co-expressed cells were enriched in the ventral VZ and decreased in the dorsal VZ (Figure 4J). Conversely, in NPC2, *PANK4*, *MMEL1*, and *PRXL2B* were positioned in scattered cells in the SVZ in both dorsal and ventral spinal cord (Figure 4K). Only a few spatial co-expression dots of *LIN72449* and *PANK4*, *MMEL1*, and *PRXL2B* were detected in scattered cells in both the dorsal and ventral spinal cord, indicating a less tight interaction of *LIN72449* with them (Figure 4L).

Our results suggest that combinations of spatial transcriptomic data and lncRNA genomic locations with coding genes can serve as a tool to predict potential *cis*-regulatory pairs of lncRNA-coding genes in the human fetal spinal cord.

### lncRNAs and *cis*-regulatory pairs in the MN trajectory

**Figure 5:**
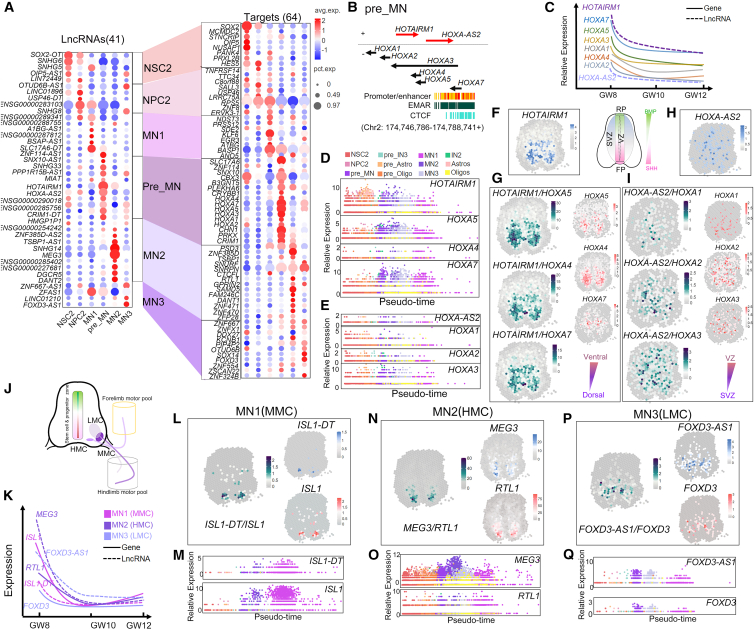
lncRNAs and their adjacent coding genes in *cis*-regulatory pairs in the motor neurons (A) Bubble charts displaying co-expressed lncRNAs and their *cis* targets in motor neuron clusters. Dot color indicates expression level (red, high; white, median; blue, low), while size shows marker gene cell fraction. (B) The *cis*-acting *HOTAIRM1* and *HOXA-AS2* in the pre_MN, with 6 genes antisense on chr2. (C) The expression trend of *HOTAIRM1*, *HOXA-AS2*, and 6 genes from GW8 to GW12. (D and E) The cell pseudo-time patterns of *HOXA4*, *HOXA5, HOXA7*, and *HOTAIRM* (D) and *HOXA1*, *HOXA3*, *HOXA3*, and *HOXA-AS2* (E) were visualized separately. (F and H) The spatial expression patterns of *HOTAIRM1* (F) and *HOXA-AS2* (H) were visualized, separately. (G and I) The co-expression patterns at *HOTAIRM1* (G), *HOXA-AS2* (I), and 6 genes. (J and K) The expression trend of 3 lncRNA gene pairs in MN1 of MMC, MN2 of HMC, and MN3 of LMC from GW8 to GW12. (L and M) The spatial expression patterns at GW10 (L) and pseudo-time trend (M) were visualized in *ISL1-DT*/*ISL1* in MN1 of MMC. (N and O) The spatial expression patterns at GW10 (N) and pseudo-time trend (O) were visualized in *MEG3*/*RTL1* in MN2 of HMC. (P and Q) The spatial expression patterns at GW10 (P) and pseudo-time trend (Q) were visualized in *FOXD3-AS1*/*FOXD3* in MN3 of LMC. Spot: 100 μm diameter.

We found that one major developmental trajectory of the NSC2 developmental lineage is to give rise to MNs. We, thus, examined whether lncRNA-coding gene *cis*-regulatory pairs exist in the MNs and identified 41 lncRNAs and 64 adjacent coding genes in the MN lineages (Figure 5A).

Interestingly, we identified two lncRNAs, *HOTAIRM1* and *HOXA-AS2*, which were associated with 6 coding genes, *HOXA1*, *HOXA2*, *HOXA3*, *HOXA4*, *HOXA5*, and *HOXA7*, in the pre_MN cluster on chr2 at p15.2, encompassing promoter/enhancer regions, an epigenetically modified accessible region (EMAR), and a CTCF (Figure 5B). In addition, *HOTAIRM1* exhibited a higher abundance than *HOXA-AS2*, even though both displayed a high-to-low expression pattern from GW8 to GW12 along the development pseudo time (Figures 5C–5E and S18B). Moreover, while *HOTAIRM1* was highly expressed in the VZ and SVZ in the ventral human fetal spinal cord, *HOXA-AS2* expressed in both dorsal and ventral spinal cord at GW8, GW10, and GW12 (Figures 5F, 5H, and S18B). The expression patterns of *HOTAIRM1* were like those of *HOXA4*, *HOXA5*, and *HOXA7*, whereas those of *HOXA-AS2* were similar to *HOXA1*, *HOXA3*, and *HOXA2* expressions (Figures 5G and 5I). These results suggest that the lncRNAs *HOTAIRM1* and *HOXA-AS2* are expressed in distinct regions with different abundances in the human fetal spinal, likely associated with their own multiple paired coding genes, which display similar expression patterns, in a *cis*-regulatory manner.

Because MNs are organized into columns in the spinal cord, we examined whether lncRNAs are also involved in this process and annotated lncRNAs expressed in the same MN clusters with known MN column markers. The lncRNA *ISL1-DT* and the coding gene *ISL* were detected in the MN1 cluster, with an enrichment in the column MMC, lnRNA *MEG3* and coding gene *RTL1* were detected in the MN2 cluster in the HMC, and lncRNA *FOXD3-AS1* and coding gene *FOXD3* were detected in the MN3 cluster in the LMC (Figures 5J–5Q). lncRNAs and their paired coding genes displayed similar expression patterns in the human fetal spinal cord, for instance, in the ventral domain, *ISL1-DT* and *ISL1* expressed in the SVZ (Figure 5L), *MEG3* and *RTL1* were broadly expressed (Figure 5N), and *FOXD3-AS1* and *FOXD3* expressed in both VZ and SVZ (Figure 5P). They also showed similar decreased expression levels from GW8 to GW12 along the developmental pseudo time (Figures 5K, 5M, 5O, 5Q, and S18B). The NSC2 lineage also gave rise to pre_IN3 and IN2 according to the trajectory analysis (Figure S19). For example, the lncRNA *GAS5* and coding genes *DARS1* and *ZBTB37* in pre_IN3, and *LHX5-AS1* and its paired coding genes *LHX5* and *SDSL* in IN2 (Figure S19). These results suggest that lncRNAs and their paired coding genes display similar expression patterns and levels in the process of MN column organization in the human fetal spinal cord.

In summary, lncRNAs and their multiple paired coding genes in the NSC2 lineage display similar expression patterns and levels in the same populations of MNs and INs, even in the same MN columns. These overlapping expressions and synchronized expression level changes suggest the existence of *cis*-regulatory modules among lncRNAs and their paired coding genes in the human fetal spinal cord.

### lncRNA *SOX2-OT* expression in the pre_Astro and pre_Oligo

**Figure 6:**
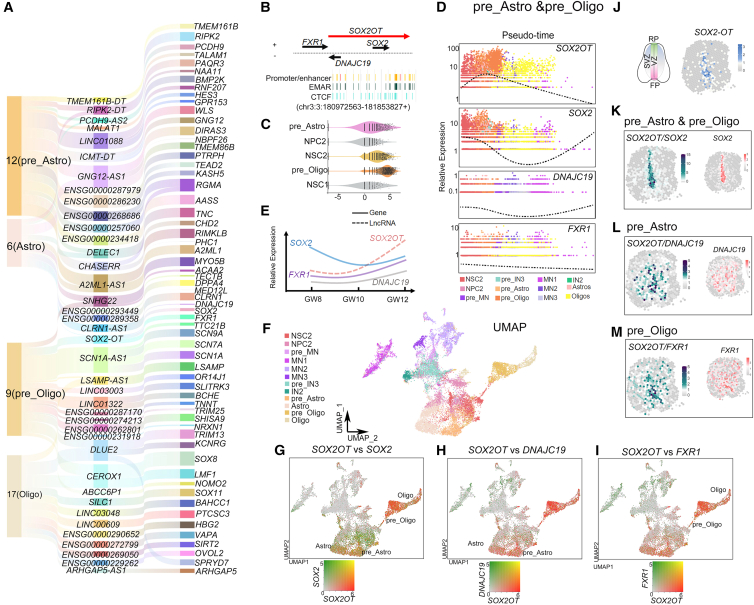
*SOX2OT* was enriched in the NCS2 to generate pre_Astro and pre_Oligo (A) Alluvial plot showing the *cis* correlation between 39 lncRNAs and 60 targets in four clusters of spinal glia development. (B) *SOX2OT* and 3 adjacent gene located in chr3. (C) The cell distribution of *SOX2OT* in NSC2, NPC2, pre_Astro, and pre_Oligo. (D) The dot plot of pseudo trend in *SOX2OT* and 3 adjacent genes, shown separately. (E) The expression trends of *SOX2OT* and 3 adjacent genes were exhibited from GW8 to GW10. (F) UMAP was visualized in NSC2 lineage. (G–I) The comparative analysis of *SOX2OT* vs. *SOX2* (G), *SOX2OT* vs. *FXR1* (H), and *SOX2OT* vs*. DNAJC19* (I), visualized by UMAP, separately (green, genes; red, *SOX2OT*; orange, co-expressed). (J–M) At GW10 spatial pattern, *SOX2OT* (J) co-expressed with *SOX2* (K), *FXR1* (L), and *DNAJC19* (M), separately. Spot: 100 μm diameter.

In the NSC2 lineage, we identified enriched expression of lncRNAs in the glial cells, including 27 (8.8%) lncRNAs in the pre_Astro cluster and 19 (6.2%) in the pre-Oligo, and annotated 39 lncRNAs and 60 potential paired coding genes in the human fetal spinal cord, as visualized using an alluvial plot (Figure 6A).

We next examined whether lncRNA-coding gene *cis*-regulatory pairs exist in the pre_Astro and pre_Oligo. Notably, we identified the lncRNA *SOX2* overlapping transcript (*SOX2-OT*), which has been previously implicated in the regulation of neurogenesis.^64^^,^^65^ *SOX2-OT* was located on chr3 q26.3 alongside SRY-box transcription factor 2 (*SOX2*) and FMR1 autosomal homolog 1 (*FXR1*), with DnaJ heat shock protein family (Hsp40) member C19 (*DNAJC19*) on the reverse strand, where it was characterized by an enrichment of promoter, enhancer, and CTCF elements (Figure 6B). Based on scRNA-seq results, *SOX2-OT* expression was elevated in NSC2 and pre_Astro clusters, with the highest expression observed in pre_Oligo clusters (Figure 6C). Throughout the pseudo time, *SOX2-OT* showed opposite expression levels to those of *SOX2, DNAJC19*, and *FXR1* from GW8 to GW12 (Figures 6D, 6E, and S18C). Further comparative analysis using UMAP revealed the co-expression of *SOX2-OT* and *SOX2* in the pre_Astro and pre_Oligo (Figures 6F and 6G). Additionally, *SOX2OT* and *DNAJC19* co-expressed in pre_Astro, while *SOX2OT* and *FXR1* co-expressed in pre_Oligo (Figures 6H and 6I).

Subsequent scStereo RNA-seq data revealed that the spatial dots for *SOX2-OT* and *SOX2* were distributed in the central canal*,* with a high intensity in the ventral region of the spinal cord at GW8, GW10, and GW12 (Figures 6J, 6K, and S18C). Co-expressions of *SOX2-OT* with *DNAJC19* in pre_Astro and with *FXR1* in pre_Oligo were predominantly observed in scattered cells in the VZ and SVZ (Figures 6L and 6M). These overlapping expressions of *SOX2-OT* with adjacent coding genes *SOX2*, *DNAJC19*, and *FXR1* imply potential *cis*-regulatory modules of the lncRNA in maintaining the NSC2 and producing precursors of Astro and Oligo.

### Abundance of lncRNAs in the Astro and Oligo trajectories

**Figure 7:**
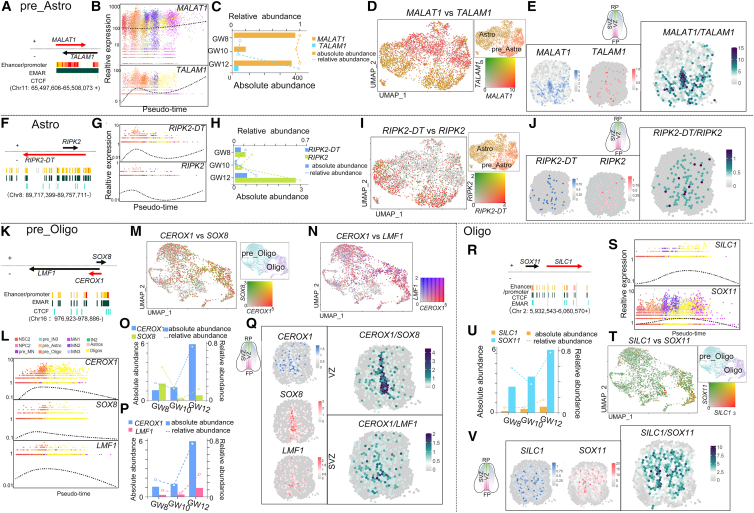
lncRNAs and their adjacent coding genes in the Astro and Oligo trajectories (A) In the cell cluster of pre_Astro, *MALAT1* and *TALAM1* were located on chr11. (B) *MALAT1* and *TALAM1* with their pseudo-time trends, shown in a dot plot. (C) A combined bar and line plot displaying their absolute and relative expressions (orange, *MALAT1*; blue, *TALAM1*). (D) UMAP was used to compare *MALAT1* and *TALAM1* expression in pre_Astro and Astro (green, *TALAM1*; red, *MALAT1*; orange, co-expressed). (E) At GW10 spatial pattern, the expressions of *MALAT1* and *TALAM1* exhibited separately. (F) In the cell cluster of Astro, *RIPK2-DT* and *RIPK2* were located on chr8. (G) *RIPK2-DT* and *RIPK2* with their pseudo-time trends shown in a dot plot in NSC2 lineage. (H) A combined bar and line plot displaying the absolute and relative expressions of *RIPK2-DT* and *RIPK2*. (I) The comparative analysis of *RIPK2-DT* and *RIPK2*, visualized by UMAP (green, *RIPK2*; red, *RIPK2-DT*; orange, co-expressed). (J) At GW10 spatial pattern, *RIPK2* and *RIPK2-DT* were exhibited, separately. (K) The *cis* lncRNA-gene pairs in the pre_Oligo: *CEROX1*, *SOX8*, and *LMF1* on chr16. (L) The dot plot of pseudo trend in *CEROX1* and 2 adjacent genes exhibited separately. (M and N) The comparative analysis of *CEROX1* vs. *SOX8* (M) and *CEROX1* vs. *LMF1* (N), visualized by UMAP in pre_Oligo and Oligo, separately. (O and P) A combined bar and line plot displaying the absolute and relative expressions of *CEROX1* vs. *SOX8* (O) and *CEROX1* vs. *LMF1* (P), separately. (Q) At GW10 spatial pattern, *CEROX1*, *SOX8*, and *LMF1* were visualized separately. (R) The *cis* lncRNA-gene pairs in the Oligo: *SLIC1* and *SOX11* on chr2. (S) The dot plot of pseudo trend in *SLIC1 and SOX11*, exhibited, separately. (T) The comparative analysis of *SLIC1* vs. *SOX11*, visualized by UMAP in pre_Oligo and Oligo. (U) A combined bar and line plot displaying the absolute and relative expressions of *SLIC1* and *SOX11*, separately. (V) At GW10 spatial pattern, *SLIC1* and *SOX11* were visualized separately. Spot: 100 μm diameter.

*MALAT1* and its adjacent lncRNA *MALAT1* antisense RNA (*TALAM1*) were located on human chr11 at q13.1, a region with enriched enhancer, promoter, and EMAR activity (Figure 7A). *MALAT1* showed high abundance at GW8 and GW12 (Figures 7B and 7C). The comparative analysis revealed that nearly all pre_Astro and Astro cells exhibit high *MALAT1* expression, with many also co-expressing *TALAM1*, suggesting an astrocyte trajectory of the lncRNA *MALAT1* (Figure 7D). Moreover, the scStereo RNA-seq data showed that *MALAT1* is highly expressed in the ventral VZ and some scattered cells in the SVZ, where it displays overlapping expression with the coding gene *TALAM1* at GW8, GW10, and GW12 (Figures 7E and S18D).

Furthermore, the lncRNA *RIPK2-DT* and its adjacent coding gene receptor interacting serine/threonine kinase 2 (*RIPK2*) were detected in the cell cluster of differentiated Astro and were located on the chr8 q21.3 in a region of enriched enhancer, promoter, and EMAR activity (Figures 7F and 7G). *RIPK2-DT* and *RIPK2* showed increased expressions from GW8 to GW12, with a similar cell distribution in both pre_Astro and Astro (Figures 7H and 7I). The spatial dots of the scStereo RNA-seq data showed that *RIPK2-DT* and *RIPK2* co-expressed in scattered cells in the dorsal and ventral regions of the human fetal spinal cord from GW8 to GW12 (Figures 7J and S18D). These results indicate that lncRNAs and adjacent genes/lncRNAs may participate in the Astro lineage specification during the human fetal development.

We next examined lncRNA expression in the Oligo trajectories. We found that lncRNA cytoplasmic endogenous regulator of oxidative phosphorylation 1 (*CEROX1*), along with the adjacent coding genes *SOX8* and lipase maturation factor 1 (*LMF1*), are located on human chr16 p13.3 in the pre_Oligo cell cluster (Figure 7K). This region was characterized by enriched promoter and EMAR activity. *CEROX1*, *SOX8*, and *LMF1* were abundant in the cell clusters of pre_Oligo and Oligo and displayed increased expression levels in the human fetal spinal cord from GW8 to GW12 (Figures 7L–7P). The scStereo RNA-seq data showed that both *CEROX1* and *LMF1* expressed in scattered cells, with a higher intensity in the ventral region in the human fetal spinal cord at GW8, GW10, and GW12 (Figures 7Q and S18D). *SOX8* was extensively expressed in the VZ in the central canal and showed overlapping expression with *CEROX1* in the ventral region, which represents expression patterns of pre_Oligo in the spinal cord (Figure 7Q).^66^^,^^67^^,^^68^

Moreover, we detected that lncRNA sciatic injury-induced lncRNA upregulator of *SOX11* (*SILC1*) and its adjacent coding gene *SOX11* are located on chr2 at p25.2 (Figure 7R). Both *SILC1* and *SOX11* exhibited predominant expression in the pre_Oligo and Oligo clusters and shared a similar increased expression in the pseudo time from GW8 to GW12 (Figures 7S and 7T). The merged spatial dots of the scStereo RNA-seq data showed that both *SILC1* and *SOX11* are highly expressed in scattered cells evenly distributed in the human fetal spinal cord, which is very similar to that of Oligo (Figures 7U, 7V, and S18D).^69^^,^^70^^,^^71^

## Discussion

The spinal cord consists of diverse neurons and glia, which are specified by complex spatial and temporal RNA transcription regulations and control sophisticated sensory perception and motor response.^72^^,^^73^ To address the spatiotemporal dynamics and cell trajectory of spinal cells, we performed and integrated the scRNA-seq and scStereo RNA-seq datasets from GW8 to GW12 in the human fetal spinal cord and characterized 3 NSC lineages at early fetus stages. In addition, we identified enriched expressions of lncRNAs and their adjacent or predicted coding genes in distinct cell types. The spatial regulations of lncRNA diversity with their associated coding genes suggest the existence of *cis*-mediated lineage switch in neurogenesis and gliogenesis of the human fetal spinal cord.

In this study, we elucidated 3 distinct NSC lineages within the human fetal spinal cord, using scRNA-seq and scStereo RNA-seq. The NSC1 cluster was identified in the dorsal spinal cord and showed a dorsal to ventral expansion, where they simultaneously produced a significant proportion of INs, likely in response to the BMP and WNT signaling pathways in the dorsal spinal cord.^57^^,^^58^^,^^59^ The NSC2 cluster was detected in the ventral spinal cord and displayed a ventral-to-dorsal expansion. It gave rise to distinct neurons, and subsequently astrocytes and oligodendrocytes. Our scRNA-seq and scStereo RNA-seq data demonstrate that distinct MN, IN, and glial subtypes are likely pre-specified in the 3 NSC clusters and, subsequently, in the progenitors in specific positions in the dorsal and ventral regions of the human fetal spinal cord.

We also identified a unique NSC cluster that maintains self-renewal capacity by expressing NSC markers such as *SOX2*, *NES*, *PAX6*, and *HES5*, which have been shown to respond to molecules including SHH, WNT, FGF, and EGF.^60^^,^^74^^,^^75^^,^^76^ It is unclear whether this NSC cluster is maintained in the adult spinal cord.^42^^,^^43^ Taking consideration of the activation of endogenous NSCs in responding to the spinal cord injuries,^8^^,^^77^^,^^78^^,^^79^^,^^80^^,^^81^ further examining the existence of this NSC cluster or other types of NSCs in the adult human spinal cord will help develop treatment strategies for spinal cord injuries.

Accumulating studies have shown that lncRNAs normally exhibit higher cell type specificity and better spatiotemporal precision than coding genes within the CNS.^46^^,^^47^ However, due to relatively low abundance of expressions in the human tissues, lncRNAs often are shadowed by coding genes in understanding neural development mechanisms.^45^ Taking the technical advantage of accuracy of revealing expression levels and positions of RNA transcripts in individual cells, driven by the scRNA-seq and scStereo RNA-seq, we, here, generated lncRNA expression profiles in the human fetal spinal from GW8 to GW12. Among 2,000 highly expressed RNA transcripts in the human fetal spinal, 381 (19.5%) were lncRNAs, suggesting a wide spread of expression of lncRNAs in humans. Moreover, these lncRNA can be annotated into distinct cell clusters, which is similar to coding genes, further demonstrating cell type-specific expression of lncRNAs. Noticeably, some lncRNAs showed unexpectedly higher expression levels than coding genes, for instance, lncRNAs *SOX2-OT*, *MALAT1*, and *HOXB-AS3*. lncRNAs with cell type-specific expressions in the spinal cells, especially those with high expression levels, make them ideal targets for further examinations of gene regulation networks in human fetal spinal cord development.

Moreover, we identified that some lncRNAs are very closely associated with their adjacent coding genes in the genome, show similar or reversed expression level changes along the fetal development, and display overlapping spatial distributions in the human fetal spinal cord, for example, the lncRNA *LIN72449* with *HES5* in the NSC2 cluster. Because *HES5* is normally expressed in NSCs and maintains the proliferating state of NSCs,^82^^,^^83^ *LIN72449* might participate in *HES5* regulation in this process. In addition, accumulating reports have demonstrated that lncRNAs often form *cis*-regulatory pairs with their adjacent coding genes, and intergenic lncRNAs function remotely with their potential target coding genes to spatially and temporally modulate gene expression.^84^^,^^85^ Judging by the similar expression patterns of *LIN72449* and *HES5* in the human fetal spinal cord, *LIN72449* might interact with *HES5* through forming a *cis*-regulatory pair.

Some lncRNAs are strongly associated with glial trajectories in the human fetal spinal cord. Previous studies have shown that *SOX2-OT* plays a role in neuronal lineage development.^64^ Interestingly, we, here, found that *SOX2-OT* displays overlapping expression with coding genes such as *DNAJC19*, which is expressed in the Astro lineage, and *FXR1*, which is in the Oligo lineage, in the human fetal spinal cord. Moreover, the lncRNA *MALAT1* has been shown to function as a decoy and to serve as a component in ribonucleoprotein (RNP) complexes in the CNS.^86^^,^^87^ We here discovered that *MALAT1* and its adjacent coding gene *TALAM1* are grouped in the Astro cluster and display overlapping expressions in the human fetal spinal cord. A previous study has demonstrated that *TALAM1* positively regulates *MALAT1* levels by promoting the 3′-end cleavage and maturation of *MALAT1* lncRNA.^88^ Thus, it is likely that *MALAT1* and *TALAM1* form a regulatory pair in modulating Astro trajectory in the human fetal spinal cord.

The SOX families have been reported to modulate the Oligo specification in both the fetus brain and spinal cord.^66^^,^^69^^,^^70^ Here, we also found the potential modulations of two lncRNAs *CEROX* and *SILC1* in regulating the spinal oligodendrocyte trajectories, in particular, *CEROX* and its adjacent coding gene *SOX8* in the oligodendrocyte precursors, and *SILC1* and *SOX11* in differentiated Oligo lineages. Moreover, regulatory interactions between *SILC1* and *SOX11* have been previously documented in the contexts of mouse memory formation and spinal neurodegenerations.^71^ Our scStereo RNA-seq data have shown that the lncRNAs *CEROX* and *SILC1* display strong overlapping expression with *SOX8* and *SOX11* in scattered cells in the human fetal spinal cord, which recaptures lncRNA expressions in the oligodendrocyte trajectories.^66^^,^^69^^,^^70^ These results imply that lncRNAs likely form a regulatory network with adjacent coding genes in specifying spinal Oligo in human glial cell development.

In summary, we have revealed molecular trajectories and spatial distributions of neuronal and glial lineages, which are derived from 3 specific NSC populations in the human fetal spinal cord, utilizing the scRNA-seq and scStereo RNA-seq from GW8 to GW12. We have generated comprehensive lncRNA expression profiles of specific neuronal and glial cell types, spatial positions in the dorsal and ventral spinal cord, and trends of expression levels along the developmental pseudo time. We also have uncovered a rich pool of lncRNA-coding genes pairs that are potentially involved in development of human fetal spinal Astro and Oligo, which will help better understand how multiple cells are specified and assembled sophistically in the human fetal spinal cord.

### Limitations of the study

In this study, we investigated the co-expressed lncRNAs and their *cis* adjacent genes in spinal neurons and glial cells from GW8 to GW12. Given the long segment of the spinal cord, we examined only the spatially co-expressed lncRNA-gene *cis* pairs at the brachial level. Future work should explore the spinal cord across various sections and developmental stages. Because of the limitation of the depth of scRNA-seq and scStereo RNA-seq, the TFs and chromatin accessibilities cannot be excluded in the lncRNA-*cis*-regulatory network. Further study of the functions of specific lncRNA-gene pairs in *cis* should be explored.

## Resource availability

### Lead contact

Further information and requests regarding resources and analyses should be directed to and will be fulfilled by the lead contact, Tao Sun (taosun@hqu.edu.cn).

### Materials availability

• This study did not generate new unique reagents.
• Due to ethical regulations and institutional policies, the availability of human samples and related materials generated in this study may be subject to restrictions. Requests for materials should be directed to the lead contact and reviewed by the Ethics Committee of Quanzhou Women’s and Children’s Hospital.

### Data and code availability

• The raw data of scRNA-seq and scStereo RNA-seq have been deposited at SRA of NCBI as the BioProject: PRJNA1347080 (https://www.ncbi.nlm.nih.gov/bioproject/PRJNA1347080).
• The processing and integration of data have been performed by Genedenovo Biotechnology Co., Ltd (Guangzhou, China) and online platform Omicsmart (http://www.omicsmart.com).
• The custom code used for statistical analysis and visualization in this study is available without restrictions in the GitHub repository at (GitHuB: https://github.com/apanhui/hsa_scRNA_SpatialRNA).
• Any additional information required to reanalyze the data reported in this article is available from the lead contact upon request.

## Acknowledgments

We are grateful for the assistance for bioinformatic analysis from the Gene Denovo Biotechnology Co., Ltd (Guangzhou, China). We are also grateful for the assistance for the preparation of graphical abstract and schematics in Figures 1A and 3E from the Figdraw 2.0 platform (https://www.figdraw.com/#/). This work was supported by the Scientific Research Funds of 10.13039/501100003815Huaqiao University (Z16Y0017 to T.S.), 16BS815 (to N.M.), and 19BS303 (to N.M.), the 10.13039/100020227Youth Innovation Foundation of Xiamen (3502Z202571029, to N.M.), the 10.13039/501100003392Natural Science Foundation of Fujian Province, China (2025J01170, to N.M.), the Fundamental Research Funds for the Central Universities (ZQN-1020, to N.M.), and the 10.13039/501100001809National Natural Science Foundation of China (32271002, to T.S.).

## Author contributions

T.S. and N.M. conceived and designed the study; N.M. performed the major experiment and bioinformatics analysis; N.M. and T.S. wrote the paper; N.M., T.S., T.L., S.-Y.H., Julianne Sun, and Jason Sun edited the paper; N.M. and T.S. designed the figures; J.W., L.C., and H.Z. performed tissue collection; N.M. and J.W. performed the sequencing analysis; N.M. and Y.S compiled the tables. All authors provided feedback.

## Declaration of interests

The authors declare no conflicts of interest.

## STAR★Methods

### Key resources table

**Table:** 

REAGENT or RESOURCE	SOURCE	IDENTIFIER
**Biological samples**		

GW8 spinal cord	–	Quanzhou Women’s and Children’s Hospital
GW10 spinal cord	–	Quanzhou Women’s and Children’s Hospital
GW12 spinal cord	–	Quanzhou Women’s and Children’s Hospital

**Deposited data**		

scRNA-seq	Nan Miao et al.	NCBI SRA: PRJNA1347080 (https://www.ncbi.nlm.nih.gov/bioproject/PRJNA1347080)
scStereo-seq	Nan Miao et al.	NCBI SRA: PRJNA1347080 (https://www.ncbi.nlm.nih.gov/bioproject/PRJNA1347080)
custom code	Nan Miao et al.	GitHub (https://github.com/apanhui/hsa_scRNA_SpatialRNA)

**Software and algorithms**		

Omicsmart	Genedenovo Biotechnology Co., Ltd (Guangzhou, China)	http://www.omicsmart.com
Stereoscope	Andersson et al.^49^	version 0.3.1 (https://github.com/almaan/stereoscope)
Cell2location	Kleshchevnikov et al.^50^	version 0.1.3 (https://github.com/almaan/stereoscope)
velocyto	La Manno et al.^51^	v0.17.13 (https://github.com/theislab/scvelo)
Cell Taxonomy	–	https://ngdc.cncb.ac.cn/celltaxonomy/
CytoTRACE	Gulati et al.^89^	R package
Slingshot	Street et al.^90^	R package
Monocle2	Qiu et al.^91^	R package
CellChat	Jin et al.^92^^,^^93^	Version 5
SCENIC	Aibar et al.^94^	R package
GENIE3	Huynh-Thu et al.^95^	R package

**Other**		

Graphical abstract	Figuredraw 2.0	https://www.figdraw.com/#/
Figure 1A	Figuredraw 2.0	https://www.figdraw.com/#/
Figure 3E	Figuredraw 2.0	https://www.figdraw.com/#/

### Experimental model and study participant details

#### Ethics committee approval and patient consent

This study was approved by the Ethics Committee of the Quanzhou Women’s and Children’s Hospital (202004). Human fetal spinal cord tissues were collected from elective pregnancy termination specimens at the Quanzhou Women’s and Children’s Hospital. The gender, ancestry, race, and origins of six spinal cord tissues (GW8, GW10 and GW12) are unspecified. Discarded human tissues from were examined only from patients who had given informed consent without any compensation. All the protocols complied with the ‘Interim Measures for the Administration of Human Genetic Resources.

#### Ethics committee approval and patient consent

Embryonic and fetal stages were extrapolated based on the date of the mother’s last menstruation, ultrasound scans of the fetus *in utero*, crown-rump length (CRL) and visual inspection. Discarded human fetal tissues were collected from GW8 to GW12. Depending on the condition and period of the procured specimens, the spinal cord was dissected. The whole spinal cord regions of interest were collected from fresh tissues and were maintained in Hibernate-E media (Thermo Fisher, A1247601).

In this study, three whole spinal cord samples at GW8, GW10 and GW12 were used for single-cell RNA-seq (scRNA-seq) analysis, and another three brachial level (the cervical and upper thoracic segments, C5-T1) at the same stages were used for single-cell Stereo RNA-seq (scStereo-seq) analysis (Table S29).

In both the scRNA-seq and scStereo-seq processes, the sample preparation, cell labeling and sequencing were all carried out in the same batch.

### Method details

#### Sample preparation for scRNA-seq and scStereo-seq

The spinal cord for scRNA-seq was dissected in fresh, cold 1× PBS and transferred to cold tissue storage solution (Miltenyi Biotec, 130-100-008, 100 mL). The spinal cord was then washed three times with 1× PBS and transferred to a digestion medium containing 2 mg/mL of collagenase IV (Gibco, 17104-019) in a hibernate-E medium (Gibco, A1247601). A pipette was used to gently and rapidly separate the tissue into small pieces. The supernatant was discarded after centrifugation at 150*g* for 3 min, and the pellet was incubated at 37 °C for 10 min in the hibernate-E medium with 1 mg/mL of papain (Shanghai Yuanye Biotechnology, S10011). To stop digestion, after papain incubation, cells were re-suspended in Hibernate-E medium. The released cells were passed through a 70 μm cell strainer (Biologix, 15–1070), collected by spinning at 500g for 5 min and re-suspended in 1× PBS and kept on ice for subsequent sequencing.

The tissues for scStereo-seq were frozen in isopentane and then embedded in OCT. The embedded tissue blocks were cryosectioned in a cryostat to generate appropriately sized sections for Visium Spatial slides while keeping the samples frozen. Sections were placed respectively on Visium Spatial Tissue Optimization Slide and Visium Spatial Slide within the capture area. HE is staining and microscope bright field imaging for sections were then processed. The Visium Spatial Tissue Optimization Slide kit was used to fit the time for permeabilization by generating fluorescently labeled cDNA tissue prints. A timer gradient was set for each capture area. Fluorescent cDNA synthesis was performed and fluorescent print of spatial positions where the cDNA reaction took place. The fluorescent print was imaged using fluorescence microscope with tissue removed. The section with the strongest fluorescence signal, minimum diffusion and longest time for permeabilization would be chosen as the fit time for permeabilization.

#### scRNA-seq

10× Genomics Cell Ranger software (version 3.1.0) was used to convert raw BCL files to FASTQ files, alignment and counts quantification. Briefly, reads with low-quality barcodes and UMIs were filtered out and then mapped to the reference genome. Reads uniquely mapped to the transcriptome and intersecting an exon at least 50% were considered for UMI counting. Cells with unusually high number of UMIs (≥8000) or mitochondrial gene percent (≥10%) were filtered out. We also excluded cells with less than 500 or more than 4000 genes detected. After removing unwanted cells from the dataset, we employed a global-scaling normalization method “LogNormalize” that normalizes the gene expression measurements for each cell by the total expression, multiplies this by a scale factor (10,000 by default), and log-transforms the results. To minimize the effects of batch effect and behavioral conditions on clustering, we used Harmony, an algorithm that projects cells into a shared embedding, in which cells are grouped by cell type rather than dataset-specific conditions, to aggregate all samples.

Integrated expression matrix is then scaled and performed on principal component analysis for dimensional reduction. Seurat implements a graph-based clustering approach. Distances between the cells were calculated based on previously identified PCs. Briefly, Seurat embed cells in a shared-nearest neighbor (SNN) graph, with edges drawn between cells via similar gene expression patterns with the reference genome version (GRCh38.p14). To partition this graph into highly interconnected quasi-cliques or communities, we first constructed the SNN graph based on the distance in PCA space and refined the edge weights between any two cells based on the shared overlap in their local neighborhoods (Jaccard distance). We then clustered cells using the Louvain method to maximize modularity. For visualization of clusters, t-distributed Stochastic Neighbor Embedding (t-SNE) were generated using the same PCs.

The scRNA-seq data was obtained by Biomarker Technologies Corporation Co. Ltd (Beijing, China), and then analyzed by Genedenovo Biotechnology Co., Ltd (Guangzhou, China). Bioinformatic analysis was performed using Omicsmart, a dynamic real-time interactive online platform for data analysis (http://www.omicsmart.com).

#### scStereo-seq

Sample fixing and imaging had been done in sample preparing and section permeabilization would be performed as follow. Permeabilization processes for the time determined by tissue optimization. The first strand of cDNA was synthesized via reverse transcription and the second strand of cDNA was synthesized via PCR. Then the cDNA is denaturation, making the second strand of cDNA dissociated from slide. The spatially barcoded, full-length cDNA was amplified via PCR to generate sufficient mass for library construction. Enzymatic fragmentation and size selection was used to optimize the cDNA amplicon size. P5, P7, i7 and i5 sample indexes, and TruSeq Read 2 (read 2 primer sequence) were added via End Repair, A-tailing, Adaptor Ligation, and PCR. The final libraries contain the P5 and P7 primers used in Illumina amplification. The Visium Spatial protocol produced Illumina-ready sequencing libraries. A Visium Spatial library comprised standard Illumina paired-end constructs which begin and end with P5 and P7. The Visium Spatial 16bp spatial barcode and 10bp UMI were encoded in Read 1, while Read 2 were used to sequence the cDNA fragment. Sample index sequences were incorporated as the i7 index read. Read 1 and Read 2 were standard Illumina sequencing primer sites used in paired-end sequencing.

Space Ranger relied on image processing algorithms to solve two key problems with respect to the slide image: deciding where tissue had been placed and aligning the printed fiducial spot pattern. Tissue detection was needed to identify which capture spots, and therefore which barcodes, would be used for analysis. Fiducial alignment was needed so Space Ranger can know where in the image an individual barcoded spot resides, since each user might set a slightly different field of view when imaging the Visium capture area. Before counting UMIs, Space Ranger attempted to correct for sequencing errors in the UMI sequences. Reads that were confidently mapped to the transcriptome were placed into groups that shared the same barcode, UMI, and gene annotation. If two groups of reads had the same barcode and gene, but their UMIs deferred by a single base, that was Hamming distance 1 apart, then one of the UMIs was likely introduced by a substitution error in sequencing. In this case, the UMI of the less supported read group was corrected to the UMI with higher support. Space Ranger again grouped the reads by barcode, UMI (possibly corrected), and gene annotation. If two or more groups of reads had the same barcode and UMI, but different gene annotations, the gene annotation with the most supporting reads was kept for UMI counting, and the other read groups are discarded. In case of a tie for maximal read supported, all read groups were discarded, as the gene could not be confidently assigned.

The scStereo RNA-seq data was obtained by Novogene Bioinformatics Technology Co. Ltd (Beijing, China), and then analyzed by Genedenovo Biotechnology Co., Ltd (Guangzhou, China).

#### Integration analysis for scRNA-seq and scStereo-seq

Each spot in spatial transcriptome contained 1–10 cells, and understanding the cell composition of each spot could improve the resolution of spatial transcriptome data and analyzed the distribution characteristics of different cell types on the slice. To improve the resolution of scStereo-seq and make scRNA-seq visible in spinal cord, we used RCTD, Stereoscope, and Cell2location tools^48^^,^^49^^,^^50^ to predict the cell composition of each spot based on the single cell transcriptome data.

RCTD^48^ is a deep learning model for image super-resolution reconstruction. RCTD (version 2.2.1, https://github.com/dmcable/spacexr) uses a reference scRNA-seq dataset with annotated cell types to deconvolve each spatial spot into cell type proportions. Briefly, RCTD fits a statistical model that estimates the mixture of cell types per spot while accounting for platform-specific effects between scRNA-seq and spatial transcriptomics technologies. The analysis was performed using the spacexr R package (2.2.1).

Stereoscope^49^ software uses deep learning models to infer the proportion of different cell types in spatial locations. The software version 0.3.1 (https://github.com/almaan/stereoscope) was used neural network models to learn the relationship between single-cell data and spatial transcriptomic data to predict the proportion of each cell type in a specific spatial location. The model was trained by minimizing the error between the predicted proportion and the actual proportion, thereby improving the accuracy of the prediction.

Cell2location^50^ (version 0.1.3, https://github.com/almaan/stereoscope) combined single-cell transcriptome data with tissue spatial information to infer the location of cell types in tissues by using Bayesian models and variable DB Bayesian inference methods. This approach could help us understand the distribution of cell types in tissues, thereby revealing the spatial structure and function of tissues. We then used heat maps, tissue maps, and circos maps to visualize the distribution of different cell types in tissues. The Integration analysis was performed by Genedenovo Biotechnology Co., Ltd (Guangzhou, China).

#### Velocito analysis

Loom files were generated for each single cell using velocyto (v0.17.13) with options -c and -U, to indicate that each BAM represents an independent cell and reads are counted instead of molecules (UMIs), respectively.^51^ The individual loom files were subsequently merged using the combine function from the loompy Python module. AnnData object was created from the h5ad file using the scvelo python module for RNA velocity analysis.^96^ Highly variable genes were identified and the corresponding spliced and un-spliced RNA counts were normalized and log_2_^transformed^ using the scvelo.pp.filter_and_normalize function. Next, the first- and second-order moments were computed for velocity estimation using the scvelo.pp.moments function. The velocities (directionalities) were computed based on the Dynamical model as defined in the scvelo.t1.velocity function, and the velocities were subsequently projected on the UMAP embeddings generated from Seurat above (https://github.com/theislab/scvelo).

#### Cell cycle removal and cell cluster annotation

Before further analysis, the cell cycle genes were excluded. The Seurat R package was used to assign a cell cycle score to each cell based on the 15 marker genes for G1 phase, 20 marker genes for S phase, 61 marker genes for G2 phase, 64 marker genes for M phase.^97^ Cells with the highest score less than 0.3 was identified as non-cycling cells.^98^

Due to the complexity of spinal cord neurons, the cell cluster annotation has been carefully considered using the online tools (Cell Taxonomy, https://ngdc.cncb.ac.cn/celltaxonomy/), which the markers have been proven and tested in humans or mice. In addition, the known genes with the correct domain of the spinal cord have been selected as the cell markers.

### Quantification and statistical analysis

#### Trajectory analysis

CytoTRACE was used to evaluate differentiation the potentials of from scRNA-seq data.^89^ We separated the major two different NSC lineages by Slingshot,^90^ and then use Monocle2 to show more specific genes in different sub-lines.^91^ In this study, we used these two methods to support our conclusion.

CytoTRACE is a non-canonical pseudo-time analysis method that does not construct differentiation trajectories but evaluates the differentiation ability of cells.^89^ The principle of CytoTRACE is show that cells at the early stages of differentiation express more genes, while those at later stages express fewer genes. CytoTRACE takes the gene expression matrix as input and predicts the cell differentiation status based on the expression patterns and the number of genes detected in the expression matrix.

Slingshot identifies lineage of cells clusters on a two-dimensional space using a minimum spanning tree (MST), determining the number of lineages and branch.^90^ Differentiation lineages have different endpoint cell clusters but share a common starting cell cluster. Subsequently, using simultaneous principal curves and orthogonal projection, Slingshot fits each differentiation lineage on a two-dimensional space and calculates the corresponding pseudo-time values for cell clusters, resulting in multiple linear cell differentiation trajectories originating from a common starting cell cluster.

The gene expression matrix is input into Monocle2, and the dimension reduction is performed. Cells are organized into a tree-like trajectory that includes branches and nodes.^91^ Subsequently, the most primitive cell clusters of differentiation state along the trajectory are defined as the cell clusters with the smallest pseudo-time value, then calculated pseudo-time values for all cells. Monocle2 defines cells along the trajectory as different differentiation states. Genes that are differentially expressed with differentiation states are filtered. The filtering threshold set to FDR<1e^−5^.

#### Cell communication and TF analysis

For the inference and analysis of cell-cell communication, we used CellChat v5, a public repository of ligands, receptors, cofactors, and their interactions.^92^^,^^93^ The expression matrix of single cell transcriptome was input, and the ligand-receptor information contained in the CellPhoneDB-data-5.0.0 database was used to calculate the percentage of cells expressing the gene and the mean value of gene expression for each gene in the cell population. Only when the proportion of cells expressing receptor and ligand genes exceeds a specified threshold (10% by default) will the ligand-receptor pair be included in the analysis. Then all cell types were randomly arranged to form a new subpopulation (1000 random arrangements by default), and the average expression abundance of receptor pairs in the cell population after random arrangement was calculated.

To carry out transcription factor network inference, analysis was performed on the SCENIC R package.^94^ In brief, log-normalized expression matrix generated using Seurat was used as input, and the pipeline was implanted in three steps. First, we identified gene co-expression network via GENIE3.^95^ Second, we pruned each module based on a regulatory motif near a transcription start site via RcisTarget. Precisely, networks were retained if the TF-binding motif was enriched among its targets, while target genes without direct TF-binding motifs were removed. The retained networks were called regulons. Third, we scored the activity of each regulon for each single cell via the AUC scores using AUCell R package.

#### cis and trans lncRNAs analysis

LncRNAs in top 2000 markers were classified into five classes according to their location relative to protein-coding genes: intergenic, bidirectional, intronic, antisense, sense and overlapping lncRNAs.^61^^,^^98^ We next analyzed the *cis* and *trans* potentials of lncRNAs and their targets in the same cluster by CyCoNP.^61^ LncRNAs is *cis*-regulation of their neighboring genes on the same allele. The up-stream lncRNAs which have intersection of promoter or other *cis*-elements may regulate gene expression in transcriptional or post-transcriptional level. The down-stream or 3’UTR region lncRNAs may have other regulatory functions. Thus, lncRNAs which had been previously annotated as “unknown region” were annotated again. LncRNAs within 100 kb up/downstream of a coding gene with absolute correlation more than 0.95 were likely to be *cis* regulators.

*Trans*-regulation of lncRNAs is co-expressed genes not adjacent to lncRNAs. We analyzed the correlation of expression between lncRNAs and protein-coding genes to identify target genes of lncRNAs. Pearson correlation coefficient was used, and protein-coding genes with absolute correlation more than 0.45. Bioinformatic analysis was performed using Omicsmart, a dynamic real-time interactive online platform for data analysis (http://www.omicsmart.com).

#### Functional analysis

Gene Ontology (GO) is an international standardized gene functional classification system which offers a dynamic-updated controlled vocabulary and a strictly defined concept to comprehensively describe properties of genes and their products in any organism. GO has three ontologies: molecular function, cellular component and biological process.

Pathway-based analysis helps to further understand genes biological functions, and KEGG is the major public pathway-related database. Pathway enrichment analysis identified significantly enriched metabolic pathways or signal transduction pathways in specific gene comparing with the whole genome background.

#### Statistical analysis

The impact of the cell cycle on cell clustering and dimensionality reduction was eliminated using the “CellCycleScoring” function, and all data were normalized using the “NormalizeData” function and “LogNormalize” method. Gene markers for cell clusters were selected from genes of differential expressions with statistical significance (adjusted *p* < 0.05) using the “FindAllMarkers” function. In the GO enrichment analysis, GO terms of genes with statistical significance (*p* < 0.05) were selected. In the Pearson correlation analyses between different cell clusters based on spatial transcriptome datasets, labeled cell clusters with correlation (correlation coefficient ≥0.7, *p* < 0.05) were calculated using the “rcorr” function in the RStudio software.

## Supplemental information

Document S1. Figures S1–S19

Table S1. Sample alignment status of scRNA-seq, related to Figure1A

Table S2. All the gene maker status with annotation of scRNA-seq, related to Figure1B

Table S3. All the genes with average PCT values of scRNA-seq, related to Figure 1B

Table S4. The cell proportion of the clusters in GW8, GW10, and GW12, related to Figure 1B

Table S5. Differentially expressed genes in the target cluster, related to Figure 1C

Table S6. The deterministic rank of velocity genes, related to Figure 1C

Table S7. The stochastic rank of velocity genes, related to Figure 1C

Table S8. The dynamical rank of velocity genes, related to Figure 1C

Table S9. Sample alignment status of Stereo RNA-seq, related to Figure 1D

Table S10. All the gene average expression with annotation of Stereo RNA-seq, related to Figure 1D

Table S11. The cluster status of Stereo RNA-seq, related to Figure 1D

Table S12. The proportion of each stack of Stereo RNA-seq, related to Figure 1D

Table S13. All gene expression with annotation details in spot analysis, related to Figure 1D

Table S14. The spot density of RTCD analysis, related to Figures 1E and 1F

Table S15. The spots values in each stack of RTCD analysis, related to Figures 1E and 1F

Table S16. The spots density of Stereoscope analysis, related to Figures 1E and 1F

Table S17. The spots values in each stack of Stereoscope analysis, related to Figures 1E and 1F

Table S18. The spots values of Cell2location analysis, related to Figures 1E and 1F

Table S19. All pathway analysis of GW8 spinal cord of Stereo RNA-seq, related to Figure 1G

Table S20. All pathway analysis of GW10 spinal cord of Stereo RNA-seq, related to Figure 1G

Table S21. All pathway analysis of GW12 spinal cord of Stereo RNA-seq, related to Figure 1G

Table S22. Cytotrace analysis of scRNA-seq, related to Figures 2A–2C

Table S23. TF analysis of scRNA-seq, related to Figure 2I

Table S24. GO analysis of scRNA-seq, related to Figure 2J

Table S25. KEGG pathway of scRNA-seq, related to Figure 2J

Table S26. *cis* lncRNA-gene pairs with gene annotation, related to Figure 3F

Table S27. *trans* correlation value of each cluster, related to Figure 3F

Table S28. *trans* lncRNA-gene pairs with gene annotation, related to Figure 3F

Table S29. Sample collection for scRNA-seq and scStereo RNA-seq, related to “Ethics committee approval and patient consent” of STAR Methods
