# Supplementary material for: Molecular signatures of cell diversity modulated by long noncoding RNAs in the human fetal spinal cord
Source: iScience. 2026 Mar 17;29(4):115399. doi: 10.1016/j.isci.2026.115399 (PMC13090696; doi:10.1016/j.isci.2026.115399)

**iScience, Volume *29***

## Supplemental information

# Molecular signatures of cell diversity modulated by long noncoding RNAs in the human fetal spinal cord

**Nan Miao, Trevor Lee, Liying Chen, Hegan Zhang, Jing Wang, Julianne Sun, Jason Sun, Yongqiang Sha, Shi-Ying Huang, and Tao Sun**

# Supplementary Figures:

**Figure S1| Velocity analysis of scRNA-seq in neural and glial related cell in developing spinal cord.**

**A.** Cell cycle phase ratios including G1, S, G2, M in each cluster of scRNA-seq in neural and glial related cell in developing spinal cord. G1: Blue, S: Green, G2: Yellow, M: Pink, non-cycling: Grey.

**B.** Pie chart used to represent the meat spliced/un-spliced ratios, and the bar chart represent the spliced/un-spliced ratios in each cluster of spinal cord NSC, NPC, precursors, neuron and glia. Blue represents spliced ratio, red represents un-spliced ratio.

**C.** The velocity length and velocity confidence visualized by UMAP, separately. From blue to red represent low to high.

**D.** Top 15 transcripts of velocity analysis visualized by heatmap in each cluster. From blue to green represent low to high.

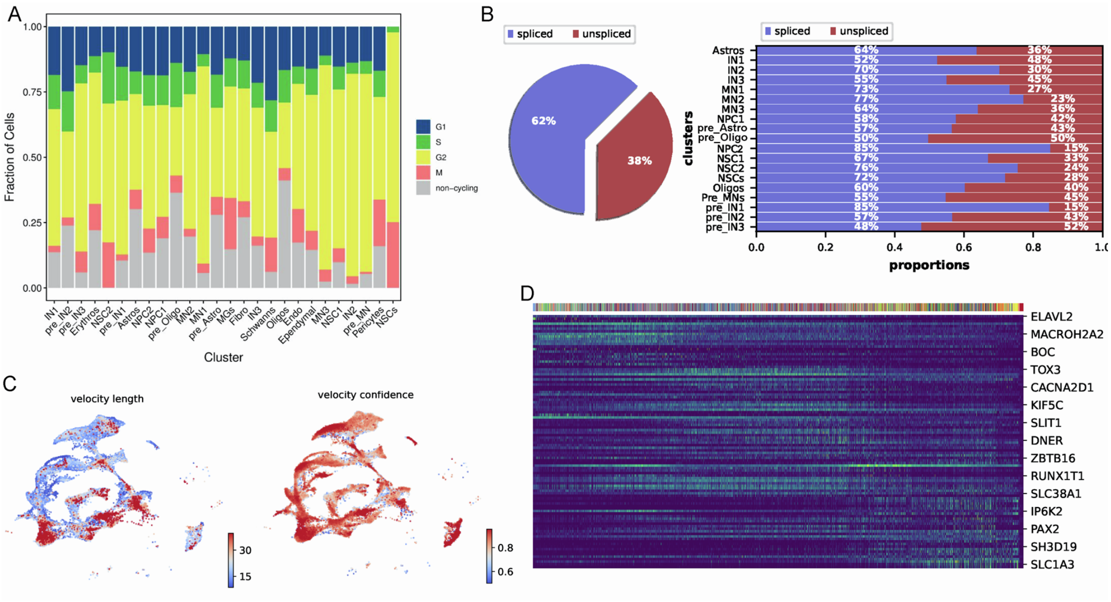

**Figure S2| The scRNA-seq and scStereo-seq of developing spinal cord from GW8 to GW10.**

**A.** The t-SNE of the 27 clusters in scRNA-seq in GW8, GW10 and GW12.

**B.** The UMAP of the 27 clusters in scRNA-seq in GW8, GW10 and GW12.

**C.** These scRNA-seq clusters were integrated into 11 scStereo-seq clusters (GW8, GW10 and GW12). The visualized spots in brachial HE slides of GW8, GW10 and GW12 spinal cord.

**D.** The t-SNE of 11 clusters in the scStereo-seq.

**E.** The UMAP of the scStereo-seq in GW8, GW10 and GW12 spinal cord.

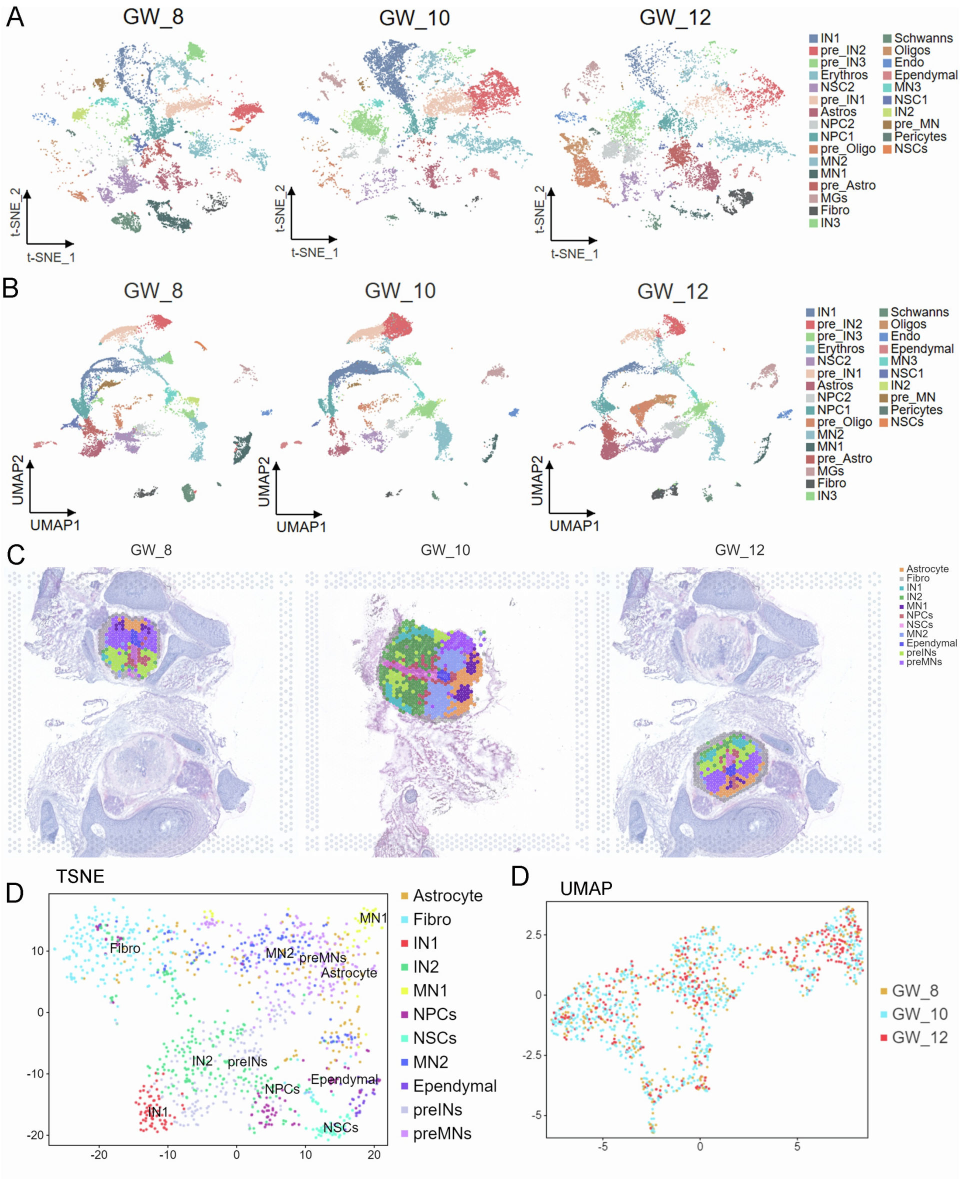

**Figure S3| The Cytotrace analysis in developing spinal cord of scRNA-seq.**

**A.** The box plots of Cytotrace scores from the cells in each cluster.

**B.** The box plots of Cytotrace scores in GW8, GW9, and GW12.

**C.**The visualized cytotrace score in UMAP. From black to pink to yellow, the cytotrace score represents more differentiated to median to less differentiated.

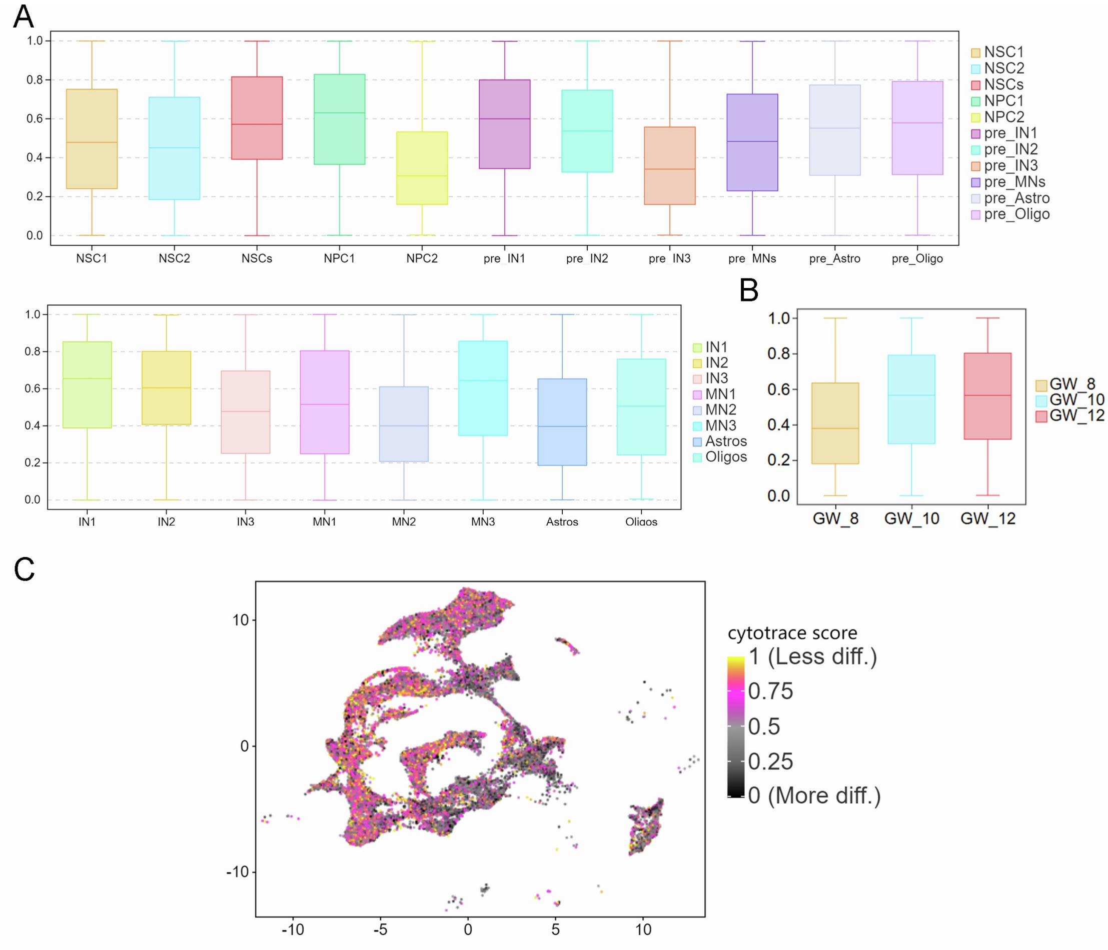

**Figure S4| The genes with spatially resolution in NSC, NPC, IN, MN, and glia cell related clusters.**

**A**. The cluster markers in NSC, NPC, IN, MN, and glia cell related clusters in GW8, GW10 and GW12 spinal cord scStereo-seq. From red to white to grey, represents the high to median to low expression. Spot: 100 μm diameter.

**B.** The KEGG analysis of the highly expressed genes in each cluster.

**C**. The bubble chart of the markers in each cluster. From red to white to grey, represents the high to median to low expression. Bubble size represents the percentage of gene expression.

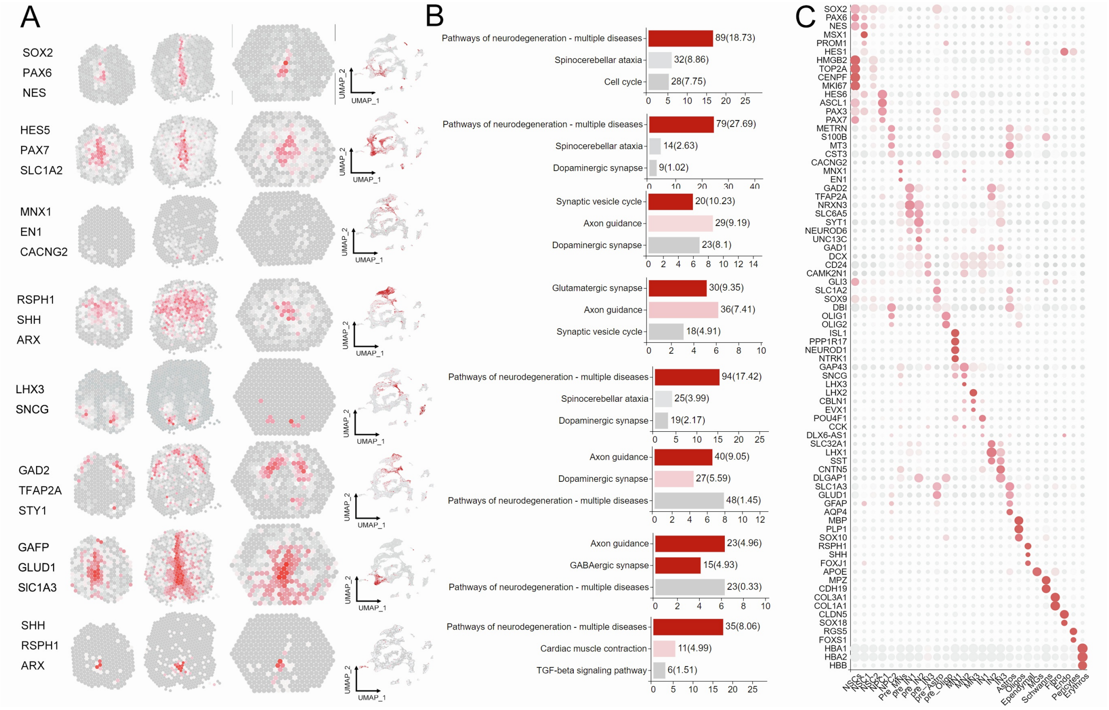

**Figure S5| The CellChat analysis in NSC1, NSC2 and NSCs.**

**A.** The cell correlation in NSC1, NSC2 and NSCs. Circles with nodes and line segments: NSCs (red), NSC2 (yellow), NSC1 (blue). The thickness of the line represents the number/expression abundance/probability by ligand receptors.

**B.** The combined bubble chart of Ligand-Receptor connection. Left bubble chart: ligands. Arrow line: connection of ligand receptor. Right bubble chart: receptors.

C. The circular chart of Ligand-Receptor connection. First circle: cluster. Second circle: ligand receptor pairs. Third circle: the expression of ligand genes (thickness) and receptor genes (arrow size).

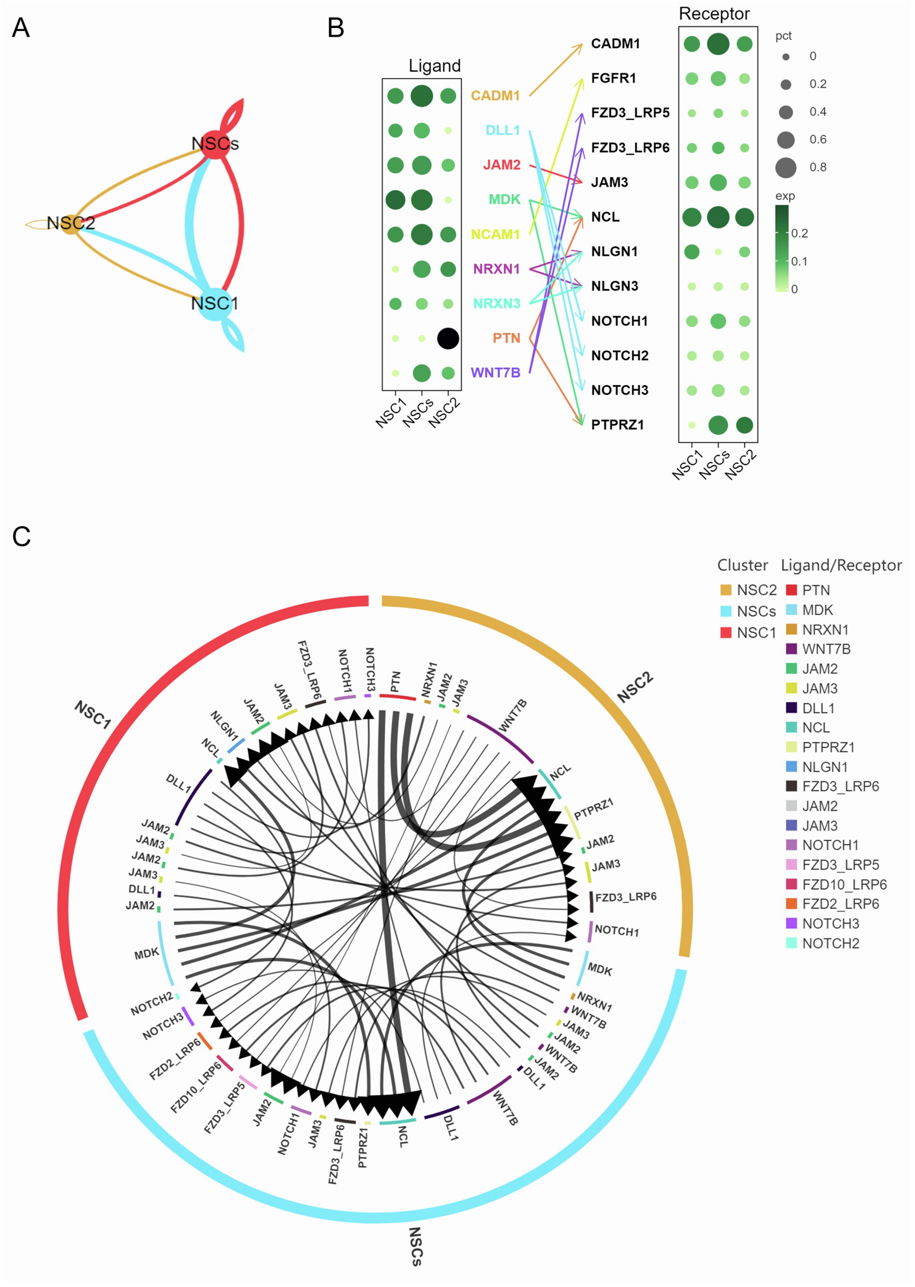

**Figure S6| The gene with visualized spatially in NPC, precursors, neurons and glia cells.**

**A.** The heatmap of cell density in NPC precursors, neurons and glia cells in GW8, GW10 and GW12.

**B.** The scStereo-seq and velocity UMAP results in NPC1, pre_IN1 and pre_IN2.

**C**. The scStereo-seq and velocity UMAP results in NPC2, pre_IN3, pre_MN, pre_Astro and pre_Oligo.

D. The scStereo-seq visualized in MN1-2, IN1-3, Astro and Oligo. Spot: 100 μm diameter.

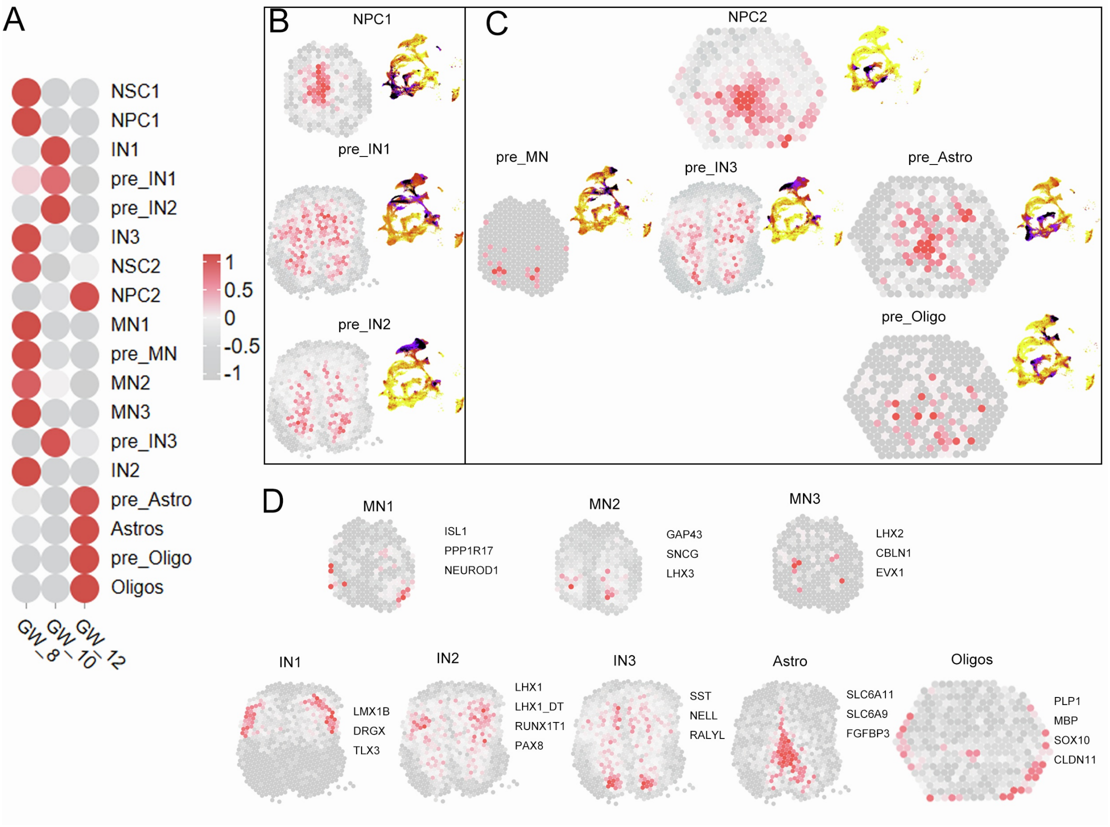

**Figure S7| The trajectory analysis of NSC1 and NSC2 lineage by Slingshot.**

**A.** The pseudo tempo analysis of the NSC1 lineage with differentiation ability in GW8, GW10 and GW12. It includes 2 sub-lineages. Lineage1: NSC1/NPC1/pre_IN1/pre_IN2/IN3. Lineage2: NSC1/NPC1/IN1.

**B.** The pseudo tempo analysis of the NSC2 lineage with differentiation ability in GW8, GW10 and GW12. It includes 4 sub-lineages. Lineage1: NSC2/NPC2/pre_Astro/Astro. Lineage2: NSC2/NPC2/pre_Oligo/Oligo. Lineage3: NSC2/NPC2/pre_IN3/IN2. Lineage4: NSC2/NPC2/MN1. Lineage4: NSC2/NPC2/pre_MN/MN2_MN3

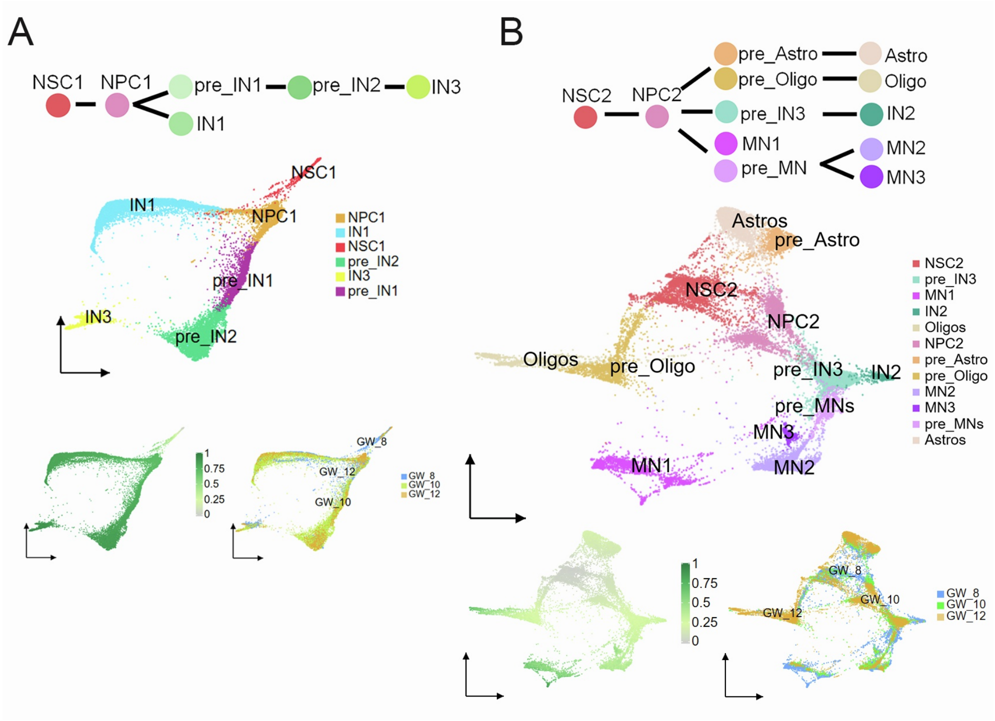

**Figure S8| The interneuron developing roles in NSC1 lineage**

**A.** The pseudo tempo analysis by Monocle 2 including cluster information with pseudo-time score in GW8, GW10 and GW12.

**B.** The genes density in NSC1 lineage, and marker genes with spatially resolution in NPC1 in GW8, IN1, pre_IN1, pre_IN2 and IN3 in GW10. Spot: 100 μm diameter.

**C.** The bubble chart of the marker genes and comparative KEGG analysis in NPC1, IN1, pre_IN1, pre_IN2 and IN3. From red to white to grey, represents the high to median to low expression. Bubble size represents the percentage of gene expression.

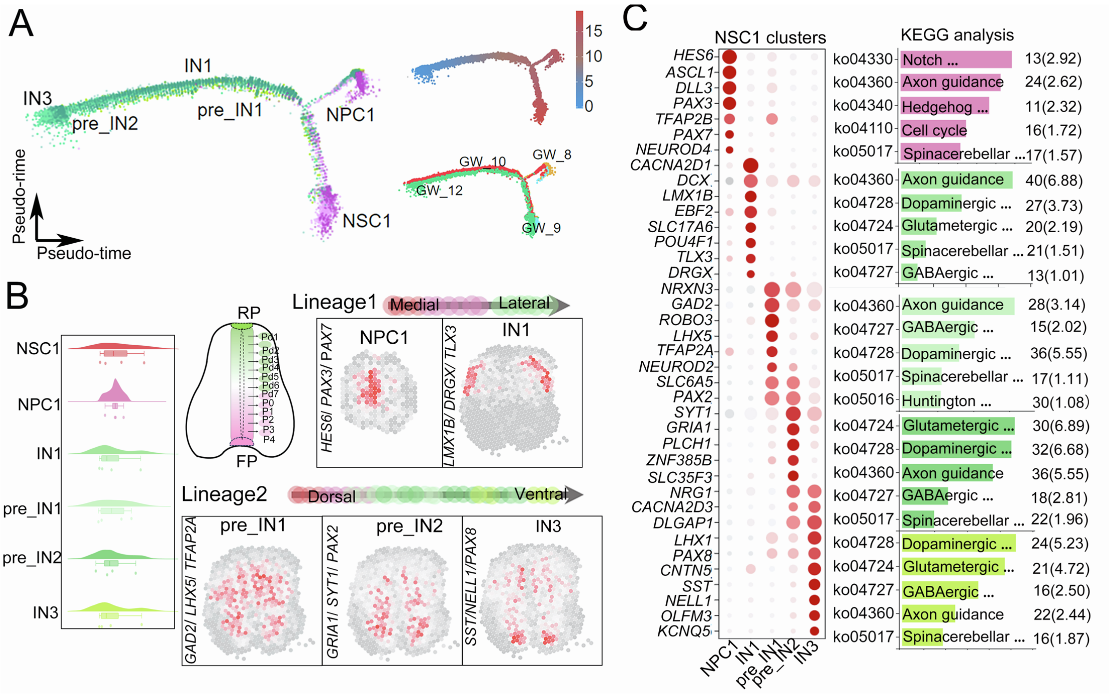

**Figure S9| The motor neuron, inter neuron and glia cell developing roles in NSC2 lineage.**

**A.** The pseudo tempo analysis by Monocle 2 with cluster information and pseudo-time score in NSC2.

**B.** The marker genes with spatially resolution in pre_MN and MN1-3 clusters in HMC, LMC and MMC in GW8.

**C.** The marker genes with spatially resolution in pre_IN3 and IN2 clusters in GW10.

**D.** The maker genes with spatially resolution in GW10, and KEGG analysis of up-regulated genes in pre_Astro and Astro clusters.

**E.** The genes with spatially resolution and KEGG analysis of up-regulated genes in pre_Oligo and Oligo clusters. Spot: 100 μm diameter.

**F.** The maker genes in heatmap of NSC2 lineages.

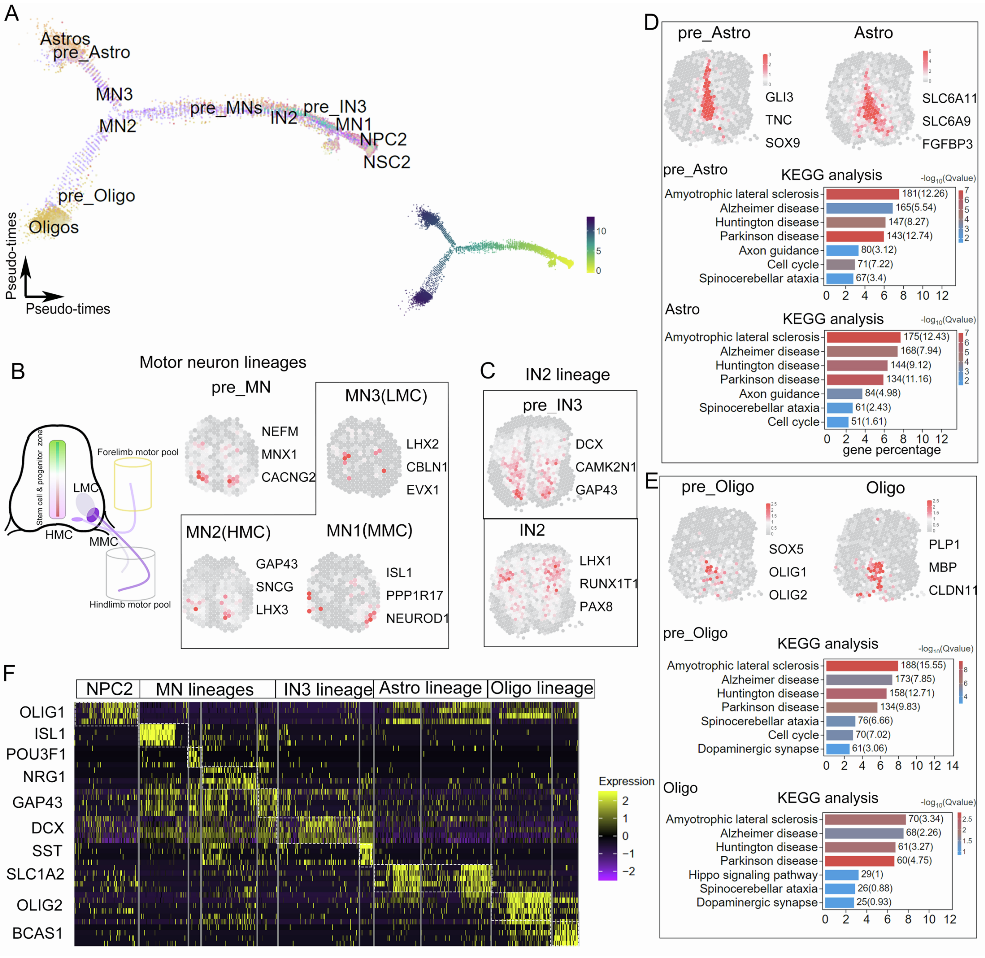

**Figure S10| The lncRNAs expression in NPC and precursor clusters visualized by UMAP.** Each dots with color represent the mean expression of two lncRNAs in a cell. From yellow to green represents the expression from low to high.

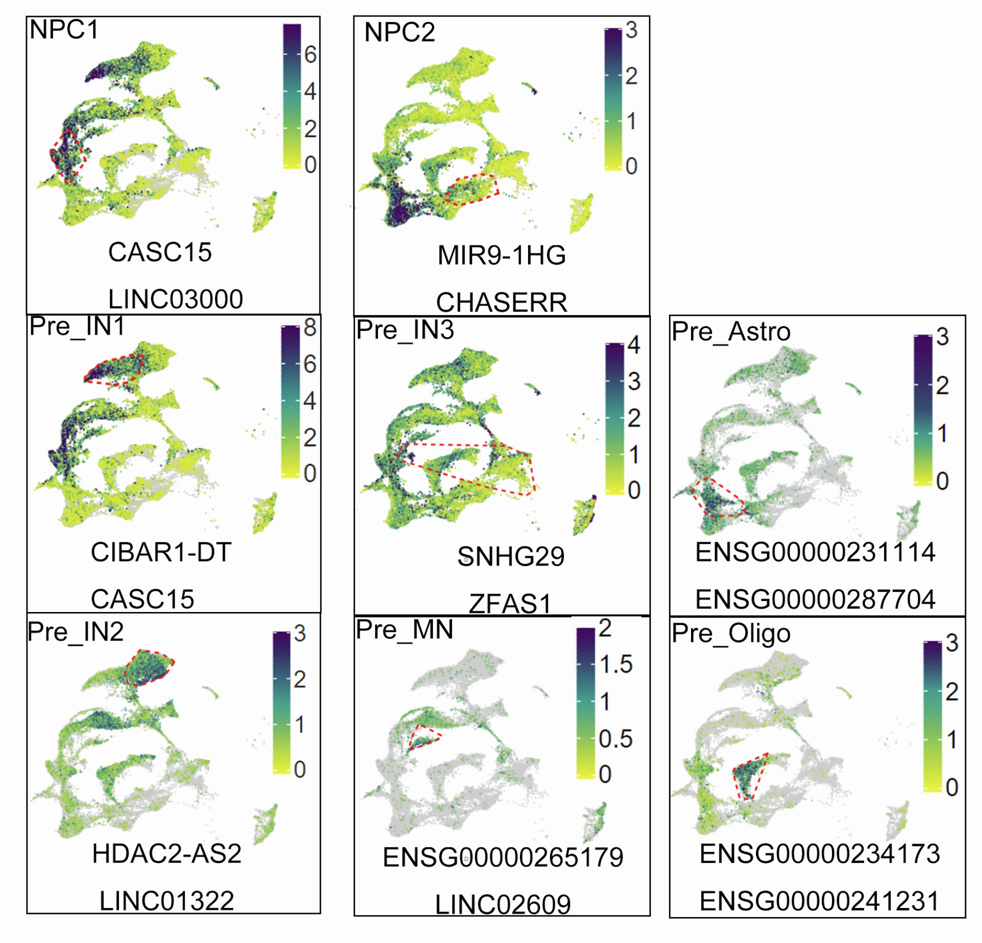

**Figure S11| The lncRNAs expression in NSC1, NSC2 and NSCs lineages visualized by scStereo-seq in GW8 and GW12.**

**A.** The lncRNA markers in 6 clusters of NSC1 lineages visualized by scStereo-seq in GW8 and GW12 (NSC1, NPC1, IN1, pre_IN1, pre_IN2 and IN3).

**B.** The lncRNA markers in 12 clusters of NSC2 lineages visualized by scStereo-seq in GW8 and GW12 (NSC2, NPC2, pre_IN3, IN3, MN1, pre_MN, MN1, MN2, pre_Astro, Astro, pre_Oligo and Oligo).

**C.** The lncRNA markers in NSCs visualized by scStereo-seq in GW8 and GW12. Spot: 100 µm diameter.

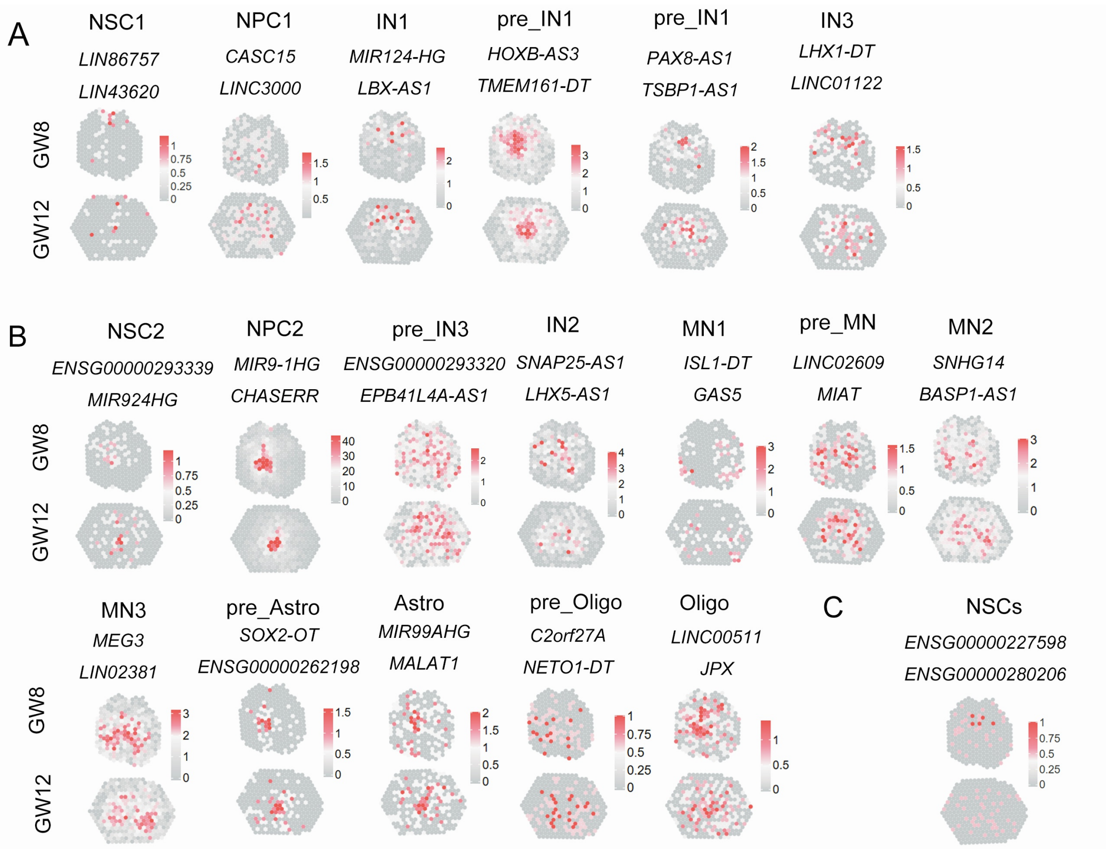

**Figure S12| The IN clusters in NSC1 lineage.**

**A.** The lncRNAs with spatially resolution of NSC1 and NPC1 are visualized in GW10 from VZ to SVZ. The heatmap represent the pseudo-tempo scores of the lncRNAs NSC1 to NPC1. From purple to yellow represents from low to high.

**B.** The lncRNAs in spatially resolution in IN1 are visualized at GW10.

**C.** The lncRNAs in spatially resolution in pre_IN1, pre_IN2 and IN3 of GW10 spinal cord. Spot: 100 μm diameter.

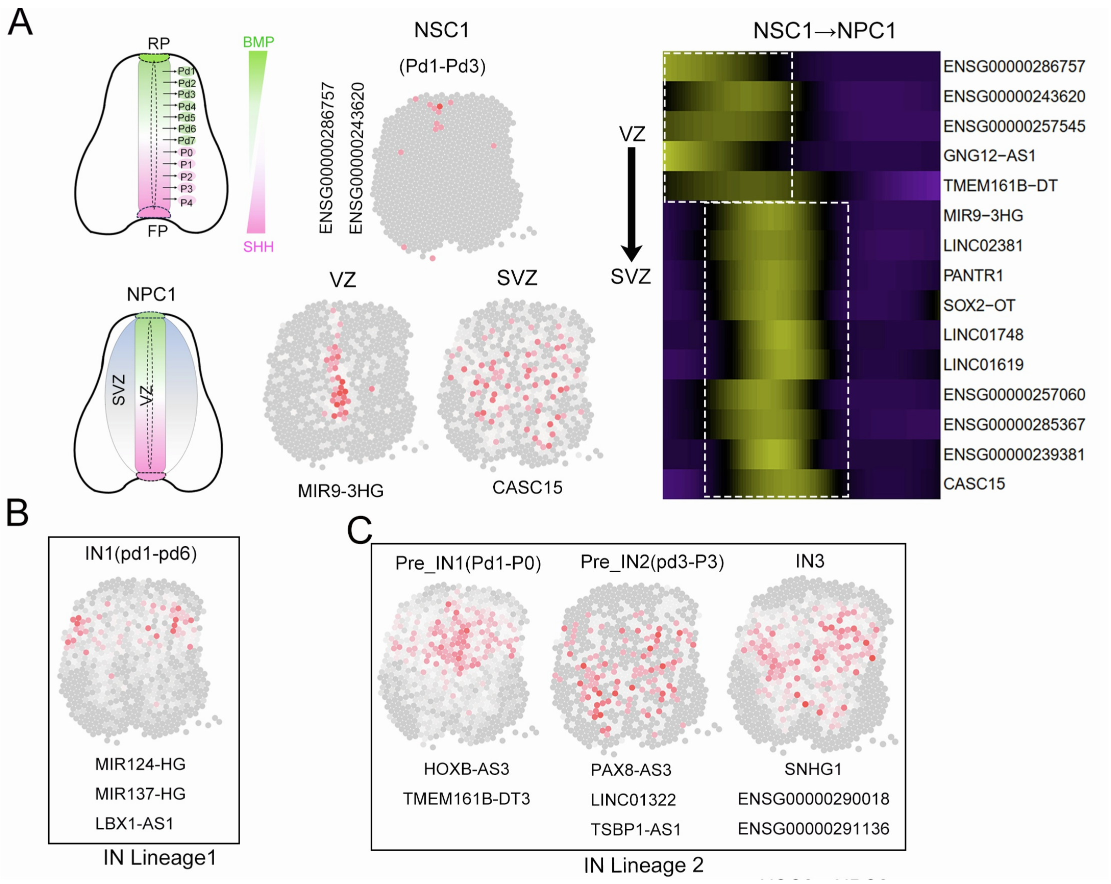

**Figure S13| The lncRNA MN clusters in NSC2 lineage.**

**A.** The pseudo tempo analysis by Monocle 2 in MN Clusters with pseudo tempo scores in GW8, GW10 and GW12. The up/down regulated genes in each cluster are visualized by bar plot and Venn gram.

**B.** The lncRNA markers with spatially resolution at GW10 were visualized in different domains: MN1 in MMC, MN2 in HMC and MN2 in LMC. Spot: 100 μm diameter.

**C.** The heatmap show the pseudo-tempo scores of the lncRNAs in pre_MN and MN1-3 clusters. From purple to yellow represents from low to high.

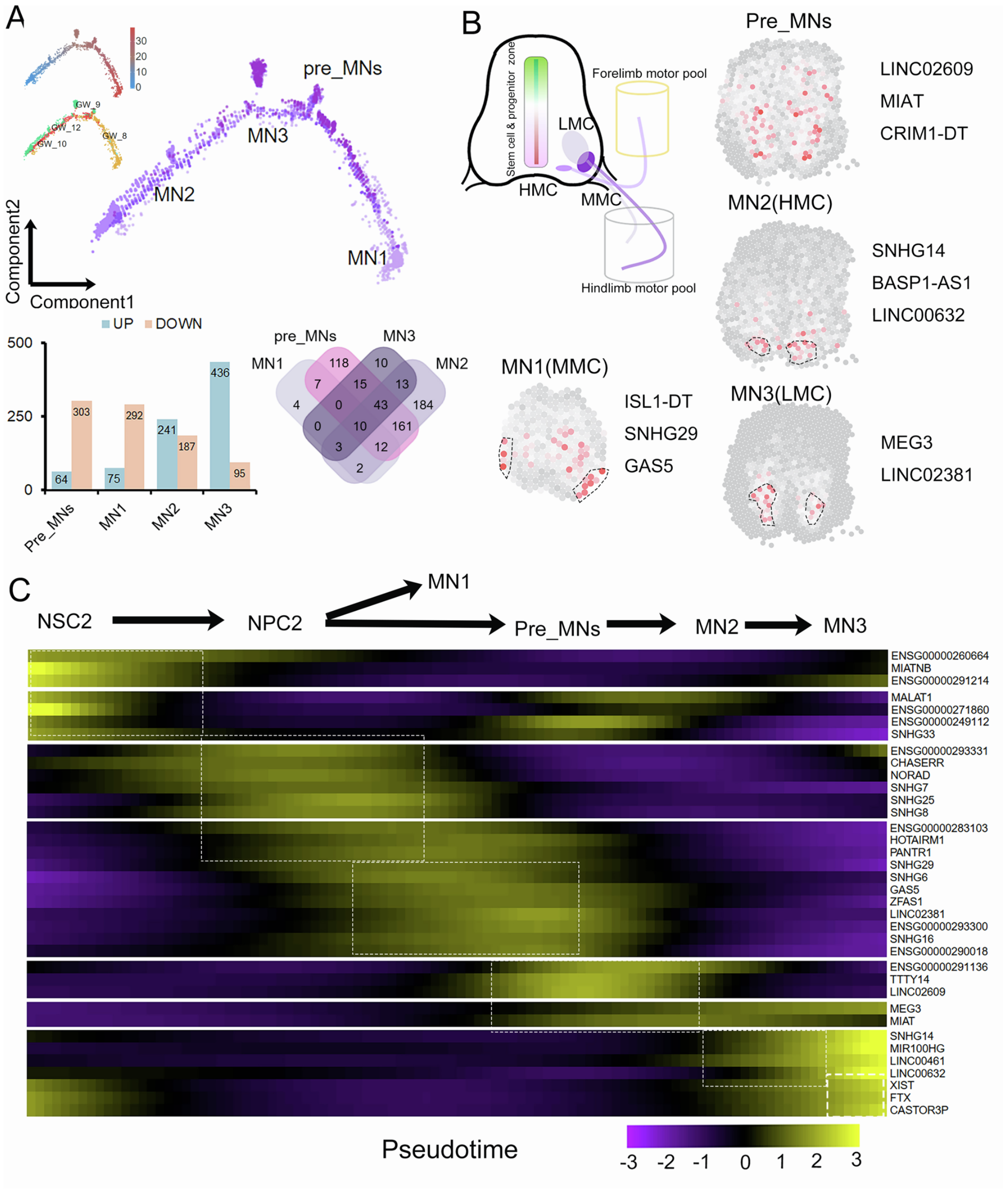

**Figure S14| The lncRNA expression in IN, astrocyte and oligodendrocyte lineages in NSC2.**

**A.** The lncRNAs with spatially resolution of NSC2 and NPC2 are visualized in GW10 from VZ to SVZ. The heatmap represent the pseudo-tempo scores of the lncRNAs NSC2 to NPC2.

**B.** The lncRNAs with spatially resolution in pre_IN3 and IN2 are visualized in GW10.

**C.** The lncRNAs with spatially resolution in pre_Astro and Astro are visualized in GW10.

**D.** The lncRNAs with spatially resolution in pre_Oligo and Oligo are visualized in GW10. Spot: 100 μm diameter.

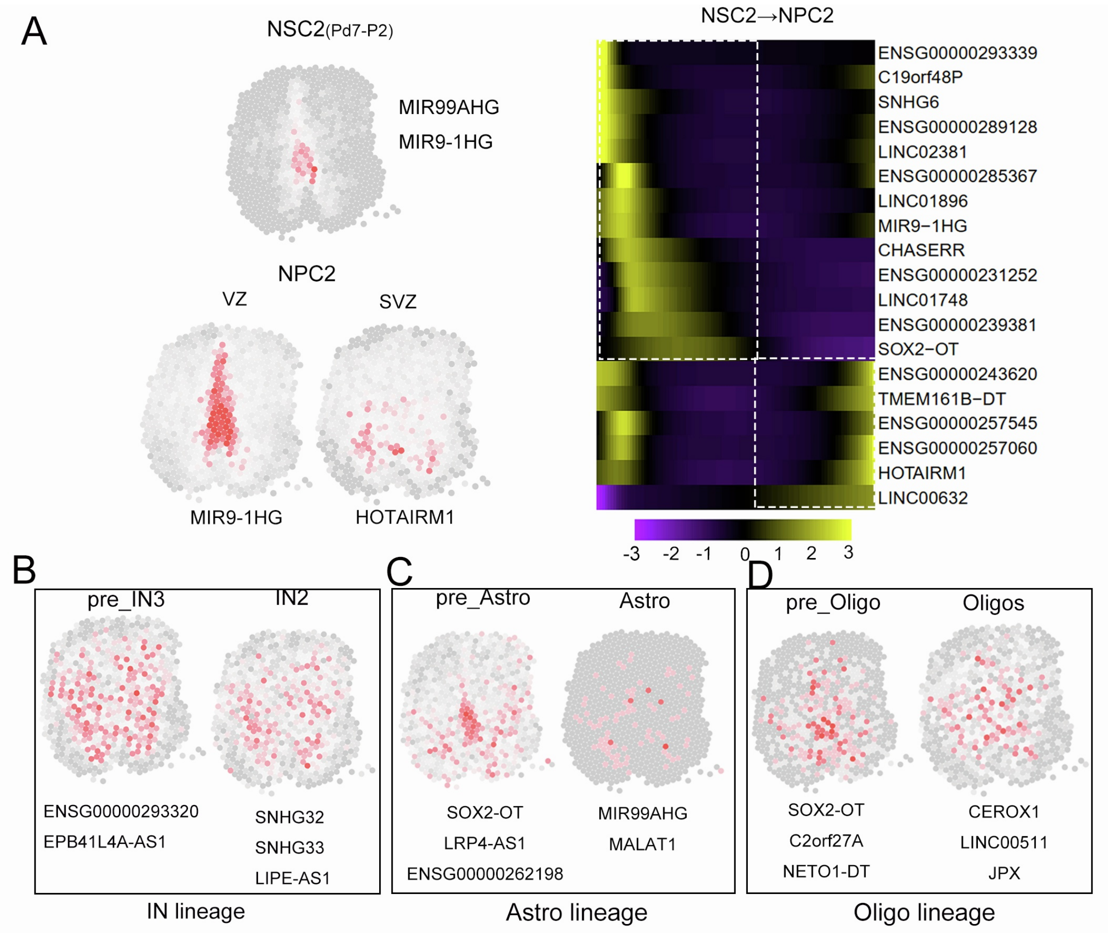

**Figure S15| The sanky plot of lncRNA-gene *cis* regulatory pairs in pre_IN1, pre_IN2, IN1 and IN3 clusters.**

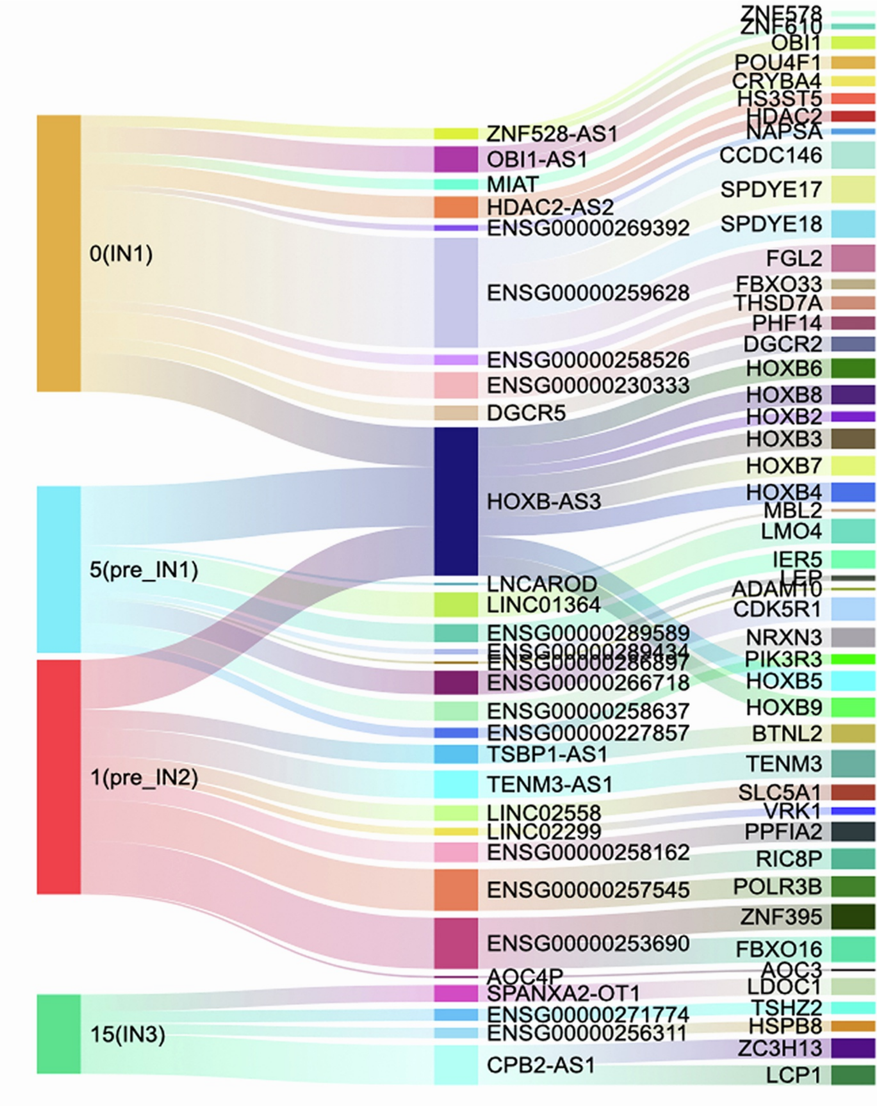

**Figure S16| LncRNAs and their adjacent coding genes in *cis*-regulatory pairs in NSC1.**

**A.** Sankey plot shows *cis* correlations between lncRNAs and targets in NSC1 and NPC1.

**B.** The *cis*-acting *LIN86757* in the NSC1 cluster is highlighted, with *ZIC2*, *ZIC5*, *CLYBL* were located on Chr.13. The expression via UMAP and spatial patterns of *LIN86757* and its targets along the dorsal-ventral axis at GW10 are shown, with circles indicating expression levels (red: high, gray: low). Expression and pseudo-time trends of these transcripts are depicted in scatter diagrams and fitted lines across six clusters via monocle2, with dots representing individual cells colored by cluster.

**C.** The *cis*-acting *LIN43620* in the NSC1 cluster, with *ZIC1* and *ZIC4* were located on Chr.3. The cell and spatial expression patterns, pseudo-time patterns of *LIN43620* and its potential *cis* targets were exhibited, separately. Spot: 100 µm diameter.

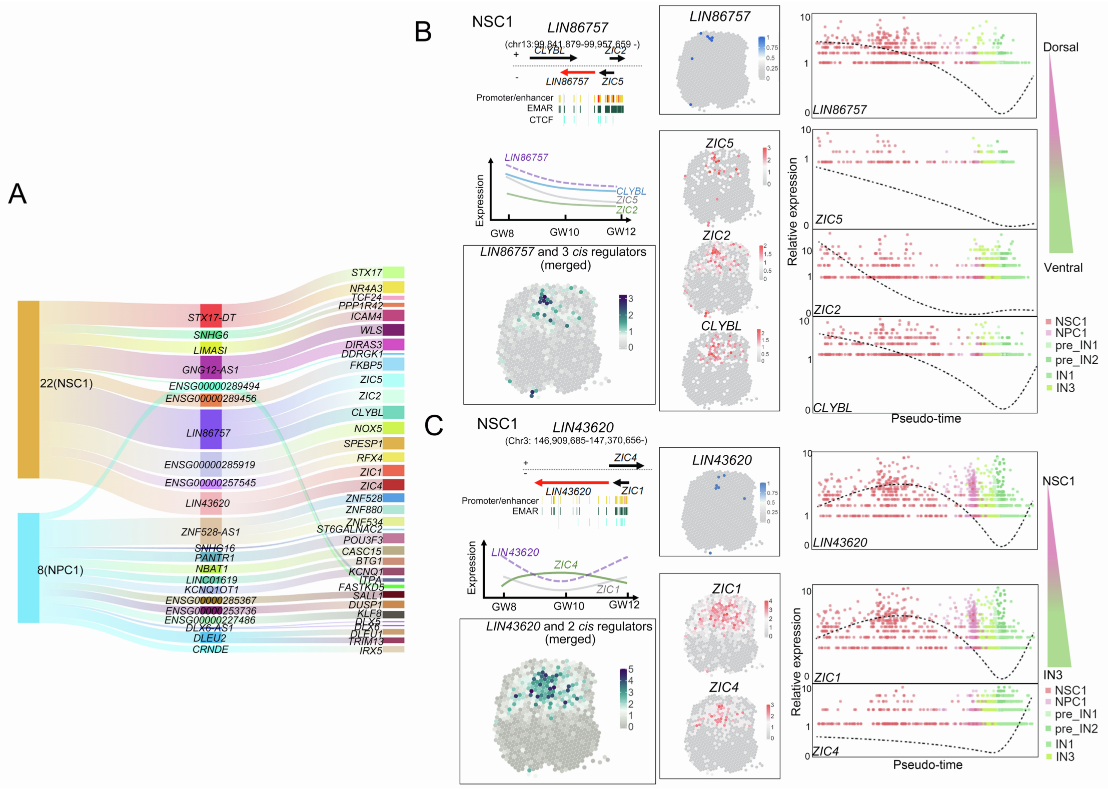

**Figure S17| LncRNAs and their adjacent coding genes in *cis*-regulatory pairs from NPC1 to IN1 and IN3.**

**A.** *PANTR1*'s *cis* role in the NPC1 cluster involves its location with *POU3F3* on Chr2. UMAP visualizes *PANTR1*'s cell expression, while spatial patterns at GW10, pseudo-time trends and expression trends from GW8-GW12 were provided.

**B.** *HOXB-AS1*'s *cis* role affects precursor and IN1 clusters, overlapping with 7 genes on Chr.17. Spatial patterns and pseudo-time trends for *HOXB-AS1*, along with *HOXB2 / 7 / 3* in pre_IN1, *HOXB9 / 5* in pre_IN2, and *HOXB6 / 8* in IN1 at GW10, are visualized. A heatmap shows the Pseudo-time trend of 8 transcripts, with expression trends from GW8-GW12.

**C.** *CPB2-AS1*'s *cis* regulatory role in IN3 overlaps with two genes on Chr.13. Visualizations include spatial patterns at GW10, Pseudo-time trends, and GW8-GW10 expression trends for *CPB2-AS1*, *ZC3H13*, and *LCP* were exhibited separately. Spot: 100 μm diameter.

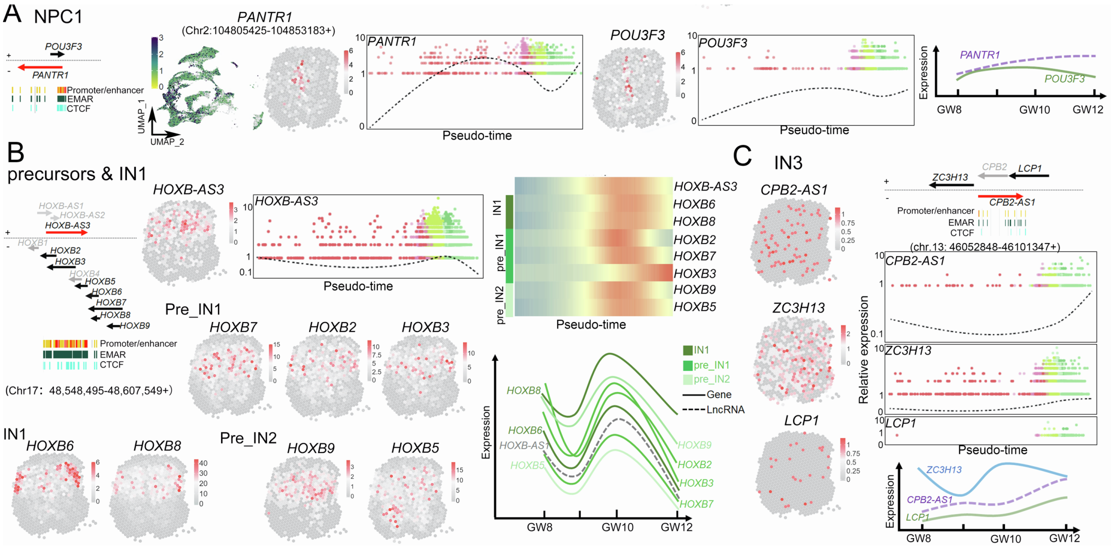

**Figure S18| The *cis* lncRNA gene pairs are visualized in GW8 and GW12**

**A.** *LIN72449* and 4 *cis* genes in NSC2 and NPC2 were visualized in GW8 and GW12.

**B.** *HOTAIRM1* and 3 *cis* genes, *HOXA-AS2* and 3 *cis* genes in pre_MN were visualized in GW8 and GW12. *ISL1-DT/ISL1* in MN1 (MMC), *MEG3/RTL1* in MN2 (HMC), *FOXD3-AS1/FOXD3* in MN3 (LMC) were visualized in GW8 and GW12.

**C.** *HOTAIRM1* and 3 *cis* genes in pre_Astro and pre_Oligo were visualized in GW8 and GW12.

**D.** *MALAT1* and *TALAM1* in pre_Astro, *RIPK2-DT* and *RIPK* in Astro, *CEROX1*, *SOX8* and *LMF1* in pre_Oligo, *SLIC1* and *SOX11* in Oligo were visualized in GW8 and GW12. Spot: 100 μm diameter.

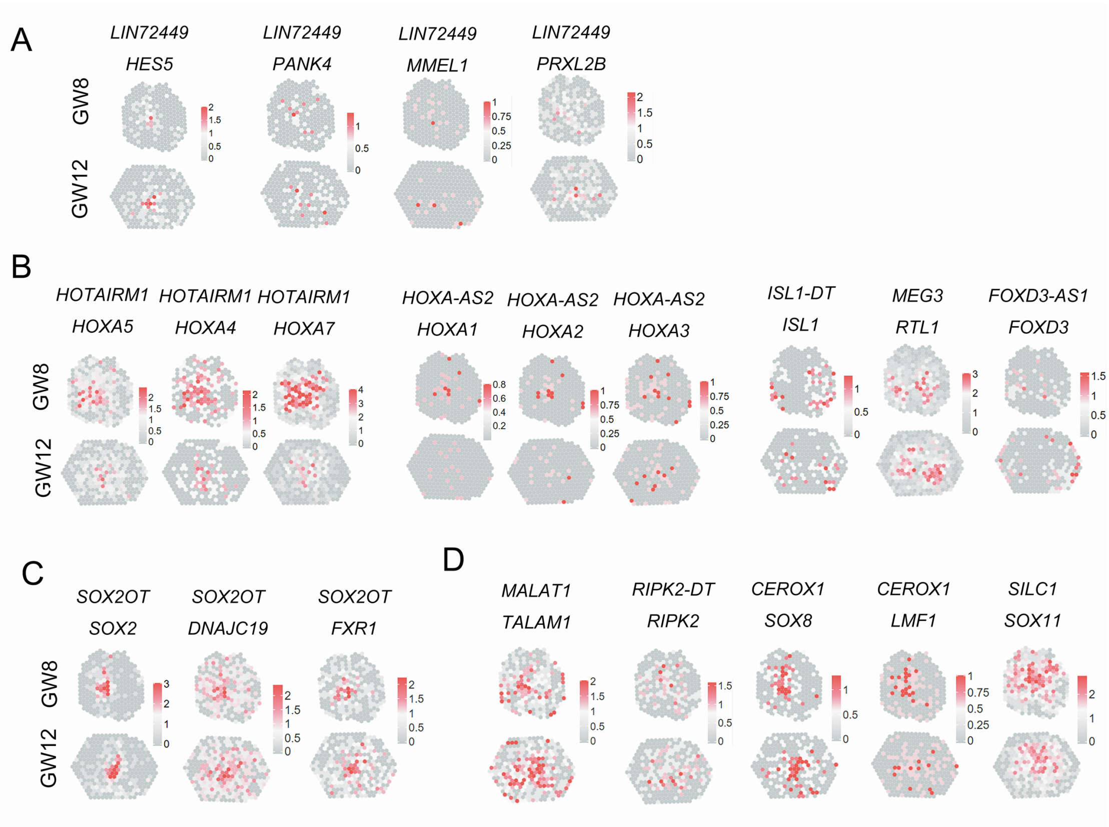

**Figure S19| LncRNAs and their adjacent coding genes in *cis*-regulatory pairs in MN1-3 and IN2.**

**A.** The spatial expression patterns at GW10 and Pseudo-time trend were visualized in IN2 clusters: *LHX5-AS1*, *LHX5* and *SDSL* in IN2, *LHX1-DT*, *LHX1* and *AATF* in IN2, separately.

**B.** Sankey plot shows *cis* correlations between lncRNAs and targets in pre_IN3 and IN2

**C.** The spatial expression patterns at GW10 and Pseudo-time trend were visualized in two IN clusters: *GAS5*, *DARS2* and *ZBTB37* in pre_IN3. Spot: 100 μm diameter.

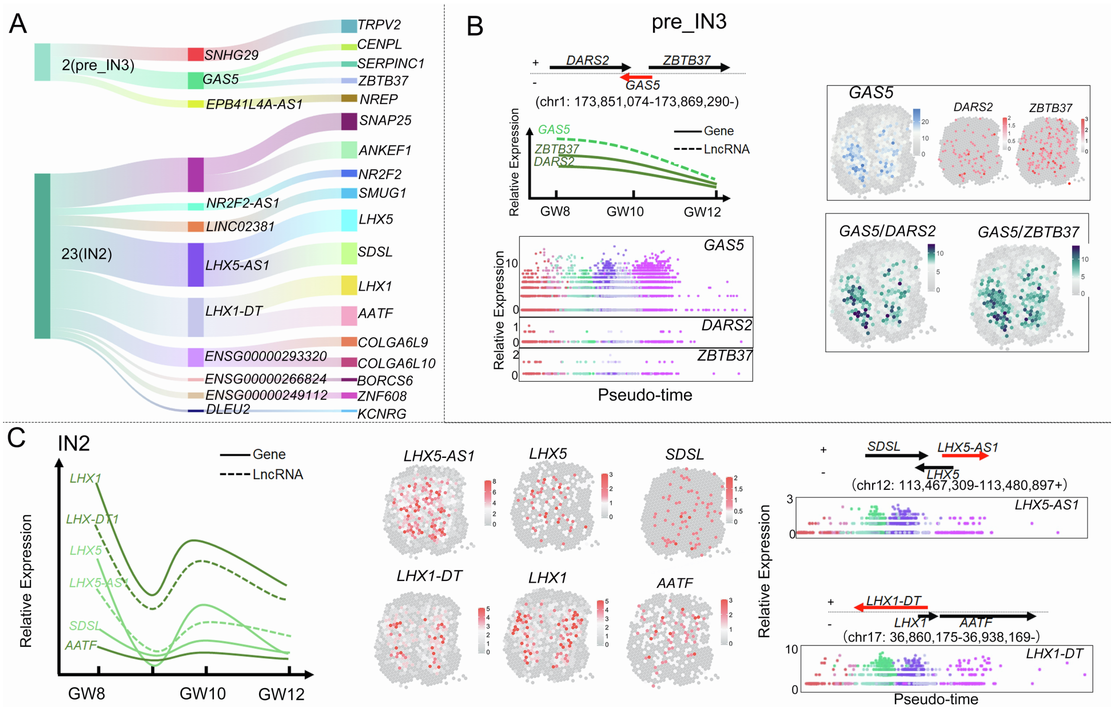

Supplement: Document S1. Figures S1–S19 [file mmc1.pdf]
